# Radical factorization in finitary ideal systems

**DOI:** 10.1080/00927872.2019.1640237

**Published:** 2019-07-26

**Authors:** Bruce Olberding, Andreas Reinhart

**Affiliations:** Department of Mathematical Sciences, New Mexico State University, Las Cruces, NM, USA

**Keywords:** Radical factorization, ideal system, monoid ring, modularization, 13A15, 13F05, 20M12, 20M13

## Abstract

In this article, we investigate the concept of radical factorization with respect to finitary ideal systems of cancellative monoids. We present new characterizations for *r*-almost Dedekind *r*-SP-monoids and provide specific descriptions of *t*-almost Dedekind *t*-SP-monoids and *w*-SP-monoids. We show that a monoid is a *w*-SP-monoid if and only if the radical of every nontrivial principal ideal is *t*-invertible. We characterize when the monoid ring is a *w*-SP-domain and describe when the *-Nagata ring is an SP-domain for a star operation * of finite type.

## 1. Introduction

The concept of factoring ideals into radical ideals has been studied by various authors. It started with papers by Vaughan and Yeagy [22, 23] who studied radical factorization in integral domains. They showed that every integral domain for which every ideal is a finite product of radical ideals (we will call such a domain an SP-domain) is an almost Dedekind domain. Later the first-named author gave a complete characterization in [17] of SP-domains in the context of almost Dedekind domains. After that the second-named author investigated the concept of radical factorization in [19, 20] with respect to finitary ideal systems. Further progress in describing SP-domains was made in [8, 13, 15]. Besides that, radical factorization in commutative rings with identity was investigated in [1]. Many of these results were extended in a recent paper [18] where radical factorization was studied in the context of principally generated *C*-lattice domains.

Ideal systems of monoids are a generalization of star operations of integral domains. They were studied in detail in [12]. It turns out that (finitary) ideal systems in general fail to be modular (i.e. the lattice of ideals induced by the ideal system is not modular). In particular, an *r*-SP-monoid (i.e. the “ideal system theoretic analogue” of an SP-domain) can fail to be *r*-almost Dedekind. The goals of this paper are manyfold. We extend the known characterizations of *r*-almost Dedekind *r*-SP-monoids for finitary ideal systems *r*. We consider lattices of ideals that are (a priori) neither principally generated nor modular. Thus we complement the results of [18] by describing the lattice of *r*-ideals in case that *r* is a (not necessarily modular) finitary ideal system. Let *p* be a modular finitary ideal system and *r* a finitary ideal system such that every *r*-ideal is a *p*-ideal. Then there is a modular finitary ideal system $\tilde{r_{p}}$ “between” *r* and *p*, called the *p*-modularization of *r*, which can be used to describe the *r*-ideals. We show, for instance, that a monoid is an $\tilde{r_{p}}$-SP-monoid if and only if every minimal prime *s*-ideal of a nontrivial *r*-finitely generated *r*-ideal is of height one and the radical of every nontrivial principal ideal is *r*-invertible. We put particular emphasis on the *t*-system and its modularizations (like the *w*-system) and present stronger characterizations for these types of ideal systems. As an application we investigate several ring-theoretical constructions with respect to the aforementioned properties.

In Section 2, we introduce the notion of (finitary) ideal systems and most of the important terminology. We also show the most basic properties of the modularizations of a finitary ideal system. In Section 3, we study finitary ideal systems in general. Our main results are characterization theorems for *r*-almost Dedekind *r*-SP-monoids as well as *r*-Bézout *r*-SP-monoids.

We put our focus on the *t*-system and its modularizations in Section 4. We will show that a monoid is a *w*-SP-monoid if and only if the radical of every nontrivial principal ideal is *t*-invertible. Moreover, we show that a monoid is both a *t*-Bézout monoid (i.e., a GCD-monoid) and a *t*-SP-monoid if and only if the radical of every principal ideal is principal.

After that we study the monoid of *r*-invertible *r*-ideals in Section 5. In particular, we characterize when every principal ideal of the monoid of *r*-invertible *r*-ideals is a finite product of pairwise comparable radical principal ideals. We also give a technical characterization of radical factorial monoids (i.e. monoids for which every principal ideal is a finite product of radical principal ideals). Furthermore, we describe when the monoid of *r*-invertible *r*-ideals is radical factorial.

Finally, we investigate several ring-theoretical constructions in Section 6. We show that if *R* is an integral domain and *H* is a grading monoid (i.e., a cancellative torsionless monoid), then R[H] is a *w*-SP-domain if and only if *R* is a *w*-SP-domain, *H* is a *w*-SP-monoid and the homogeneous field of quotients of R[H] is radical factorial. We also show that if * is a star operation of finite type of an integral domain *R*, then the *-Nagata ring of *R* is an SP-domain if and only if *R* is a *-almost Dedekind *-SP-domain.

## 2. Ideal systems

In this section, we introduce the notion of (finitary) ideal systems and the most important terminology. In the following, a monoid *H* is always a commutative semigroup with identity and more than one element such that every nonzero element of *H* is cancellative. If not stated otherwise, then *H* is written multiplicatively.

Throughout this paper let H be a monoid and let G be the quotient monoid of H.

Let *z*(*H*) denote the set of *zero elements* of *H* (i.e., z(H)={z∈H|zx=z for all x∈H}). (We introduce this notion to handle both monoids with and without a zero element. Also note that |z(H)|≤1.)

Let X⊆H and Y⊆G. Set $\sqrt{X}=\{x\in H|x^{n}\in X$ for some n∈N}, called the *radical* of *X* and $Y^{-1}=\{z\in G|\mathrm{zY}\subseteq H\}.$ We say that *X* is an *s-ideal* of *H* if X=XH∪z(H) and we say that *X* is *radical* if $\sqrt{X}=X.$ An *s*-ideal *J* of *H* is called a *principal ideal* of *H* if it is generated by at most one element (i.e., J=AH∪z(H) for some A⊆H with |A|≤1).

If a∈H, then *a* is called *prime (primary, radical)* if *aH* (i.e. the principal ideal generated by *a*) is a prime (primary, radical) *s*-ideal of *H*. Let X(H) denote the set of minimal prime *s*-ideals of *H* which properly contain *z*(*H*) and let P(X) denote the set of prime *s*-ideals of *H* that are minimal above *X*.

By $H^{•}$ (resp. $H^{\times }$) we denote the set of nonzero elements of *H* (resp. the set of units of *H*) and by P(H) we denote the power set of *H*. Let $r:P(H)\to P(H),X\mapsto X_{r}$ be a map. For subsets X,Y⊆H and c∈H we consider the following properties:

- $\mathrm{XH}\cup z(H)\subseteq X_{r}.$
- If $X\subseteq Y_{r},$ then $X_{r}\subseteq Y_{r}.$
- $cX_{r}={\left(\mathrm{cX}\right)}_{r}.$
- $X_{r}=\underset{E\subseteq X,|E|<\infty }{\cup }E_{r}.$

We say that *r* is a *(finitary) ideal system* on *H* if *r* satisfies properties *A*, *B*, *C* (and *D*) for all X,Y⊆H and c∈H. Also note that an ideal system *r* is finitary if and only if $X_{r}\subseteq \underset{E\subseteq X,|E|<\infty }{\cup }E_{r}$ for all X⊆H. Furthermore, if *r* is an ideal system, then it follows from (A) and (B) that *r* is idempotent (i.e., ${\left(X_{r}\right)}_{r}=X_{r}$ for each X⊆H).

Let *r* be finitary ideal system on *H* and X⊆H. We say that *X* is an *r-ideal* (resp. an *r-invertible r-ideal*) if *X_r_* = *X* (resp. if *X_r_* = *X* and ${\left(XX^{-1}\right)}_{r}=H$). Now let *I* be an *r*-ideal of *H*. The *r*-ideal *I* is called *nontrivial* if z(H)⊊I and it is called *proper* if I⊊H. By $I_{r}(H)$ (resp. $I_{r}^{*}(H)$) we denote the set of *r*-ideals (resp. the set of *r*-invertible *r*-ideals) of *H*. Observe that $\sqrt{I}=\underset{P\in P(I)}{\cap }P$ and $P(I)\subseteq I_{r}(H).$ If *I* and *J* are *r*-ideals of *H*, then ${\left(\mathrm{IJ}\right)}_{r}$ is called the *r-product* of *I* and *J*. Note that the set of *r*-ideals forms a commutative semigroup with identity under *r*-multiplication and the set of *r*-invertible *r*-ideals of *H* forms a monoid under *r*-multiplication.

Note that every (nontrivial) principal ideal of *H* is an (*r*-invertible) *r*-ideal of *H*. Let H be the set of nontrivial principal ideals of *H*, $q(I_{r}^{*}(H)),$ resp. q(H), the quotient group of $I_{r}^{*}(H),$ resp. H, and $C_{r}(H)=q(I_{r}^{*}(H))/q(H),$ called the *r-class group* of *H*. Note that $C_{r}(H)$ is trivial if and only if every *r*-invertible *r*-ideal of *H* is principal. Moreover, $C_{r}(H)$ is torsionfree if and only if for all k∈N and $I\in I_{r}^{*}(H)$ such that ${\left(I^{k}\right)}_{r}$ is principal, it follows that *I* is principal. Let *r*-spec(H), resp. *r*-max(H) denote the set of prime *r*-ideals, resp. the set of *r*-maximal *r*-ideals of *H*. We say that $I\in I_{r}(H)$ is *r*-finitely generated if *I* = *E_r_* for some finite E⊆I.

We say that *r* is *modular* if for all *r*-ideals *I*, *J*, *N* of *H* with I⊆N it follows that ${\left(I\cup J\right)}_{r}\cap N\subseteq {\left(I\cup (J\cap N)\right)}_{r}$ (equivalently, for all *r*-ideals *I*, *J*, *N* of *H* with I⊆N it follows that ${\left(I\cup J\right)}_{r}\cap N={\left(I\cup (J\cap N)\right)}_{r}$). Now let *p* be a finitary ideal system on *H*. The ideal system *p* is called finer than *r* (or *r* is called coarser than *p*), denoted by p≤r, if $X_{p}\subseteq X_{r}$ for all X⊆H (equivalently, every *r*-ideal of *H* is a *p*-ideal of *H*). The notions of finer and coarser can be extended to arbitrary ideal systems.

Next we introduce the most important ideal systems. Let $T\subseteq H^{•}$ be multiplicatively closed (i.e., 1∈T and xy∈T for all x,y∈T). Then there is a unique finitary ideal system *T*^−1^*r* defined on *T*^−1^*H* such that $T^{-1}(X_{r})={\left(T^{-1}X\right)}_{T^{-1}r}$ for all X⊆H. Furthermore, if *r* is modular, then *T*^−1^*r* is modular. If *P* is a prime *s*-ideal of *H*, then we set $r_{P}={\left(H\setminus P\right)}^{-1}r.$ First we define the *s*-system.

Let s:P(H)→P(H),X↦XH∪z(H).

Note that *s* is a finitary ideal system on *H*. Next we introduce the *v*-system and the *t*-system. First let *H* =* G*.

$$\text{For }X\subseteq H\text{ let }X_{v}=X_{t}=z(H)\text{ if }X\subseteq z(H)\text{ and }X_{v}=X_{t}=H\text{ if }X⊈z(H)\text{.}$$

Now let H=G.

$$\text{Let }v:P(H)\to P(H),X\mapsto {\left(X^{-1}\right)}^{-1}\text{ and }t:P(H)\to P(H),X\mapsto \underset{E\subseteq X,|E|<\infty }{\cup }E_{v}.$$

A subset *A* of *H* is called a divisorial ideal if *A_v_* = *A*. Note that every *r*-invertible *r*-ideal of *H* is divisorial. Let *R* be an integral domain. Now we define the *d*-system.

$$\text{Let }d:P(R)\to P(R),X\mapsto_{R}(X)\text{,}$$

where ${}_{R}(X)$ is the ring ideal generated by *X*. Now let p≤r. Next we introduce a finitary ideal system $\tilde{r_{p}}$ depending on *p* and *r*. We study some of its elementary properties in Lemma 2.1.

$$\text{Let }{\tilde{r}}_{p}:P(H)\to P(H),X\mapsto \{x\in H|xF\subseteq X_{p}\text{ and }F_{r}=H\text{ for some }F\subseteq H\}.$$

Lemma 2.1. *Let p and r be finitary ideal systems on H such that* p≤r.

1. $\tilde{r_{p}}$ *is a finitary ideal system on H such that* $p\leq \tilde{r_{p}}\leq r.$
2. *r-*$\max(H)=\tilde{r_{p}}$*-*max(H) *and* $X_{{\tilde{r}}_{p}}=\cap_{M\in r\text{-}\max(H)}{\left(X_{p}\right)}_{M}$ *for each* X⊆H.
3. $I_{r}^{*}(H)=I_{\tilde{r_{p}}}^{*}(H)$*, as monoids.*
4. *If p is modular, then* $\tilde{r_{p}}$ *is modular.*
5. *If m and n are finitary ideal systems on H such that* $p\leq m\leq \tilde{r_{p}}\leq n\leq r$*, then* $\tilde{n_{m}}=\tilde{r_{p}}.$

Proof.

1. Claim 1. If Y⊆H and *N* is a finite subset of $Y_{\tilde{r_{p}}},$ then $\mathrm{NF}\subseteq Y_{p}$ and *F_r_* = *H* for some F⊆H.
  Let Y⊆H and let *N* be a finite subset of $Y_{\tilde{r_{p}}}.$ For each e∈N, there is some subset *F_e_* of *H* such that $eF_{e}\subseteq Y_{p}$ and ${\left(F_{e}\right)}_{r}=H.$ Set $F=\prod_{e\in N}F_{e}.$ Then $\mathrm{NF}\subseteq Y_{p}$ and *F_r_* = *H*. □(Claim 1)
  Claim 2. If X⊆H and $x\in X_{\tilde{r_{p}}},$ then there are some finite E⊆H and some finite N⊆X such that $\mathrm{xE}\subseteq N_{p},$ *E_r_* = *H*, $x\in N_{\tilde{r_{p}}}$ and $x\in X_{r}.$
  Let X⊆H and $x\in X_{\tilde{r_{p}}}.$ There is some E⊆H such that $\mathrm{xE}\subseteq X_{p}$ and *E_r_* = *H*. Since *r* is finitary, we can assume without restriction that *E* is finite. Since *p* is finitary, there is some finite N⊆X such that $\mathrm{xE}\subseteq N_{p}.$ Consequently, $x\in N_{\tilde{r_{p}}}$ and $x\in \mathrm{xH}=xE_{r}={\left(\mathrm{xE}\right)}_{r}\subseteq {\left(X_{p}\right)}_{r}=X_{r}.$ □(Claim 2)
  Let X,Y⊆H and c∈H. If $y\in X_{p},$ then since ${\left\{1\right\}}_{r}=H$ and $y\in y\{1\}\subseteq X_{p},$ we have that $y\in X_{\tilde{r_{p}}}.$ Therefore, $X_{p}\subseteq X_{\tilde{r_{p}}},$ and hence $X_{s}\subseteq X_{\tilde{r_{p}}}.$
  Next we show that if $X\subseteq Y_{\tilde{r_{p}}},$ then $X_{\tilde{r_{p}}}\subseteq Y_{\tilde{r_{p}}}.$ Let $X\subseteq Y_{\tilde{r_{p}}}$ and $x\in X_{\tilde{r_{p}}}.$ By Claim 2 there are some finite E⊆H and some finite N⊆X such that $\mathrm{xE}\subseteq N_{p}$ and *E_r_* = *H*. By Claim 1 there is some F⊆H such that $\mathrm{NF}\subseteq Y_{p}$ and *F_r_* = *H*. This implies that $\mathrm{xEF}\subseteq N_{p}F\subseteq {\left(N_{p}F\right)}_{p}={\left(\mathrm{NF}\right)}_{p}\subseteq Y_{p}$ and ${\left(\mathrm{EF}\right)}_{r}=H,$ and hence $x\in Y_{\tilde{r_{p}}}.$
  Now we show that $cX_{\tilde{r_{p}}}={\left(\mathrm{cX}\right)}_{\tilde{r_{p}}}.$ First let $z\in X_{\tilde{r_{p}}}.$ There is some E⊆H such that $\mathrm{zE}\subseteq X_{p}$ and *E_r_* = *H*. Since $\mathrm{czE}\subseteq cX_{p}={\left(\mathrm{cX}\right)}_{p}$ and *E_r_* = *H*, we have that $\mathrm{cz}\in {\left(\mathrm{cX}\right)}_{\tilde{r_{p}}}.$ Therefore, $cX_{\tilde{r_{p}}}\subseteq {\left(\mathrm{cX}\right)}_{\tilde{r_{p}}}.$
  Now let $z\in {\left(\mathrm{cX}\right)}_{\tilde{r_{p}}}.$ It follows by Claim 2 that $z\in {\left(\mathrm{cX}\right)}_{r}=cX_{r},$ and hence *z* = *cv* for some v∈H. If c∈z(H), then $z\in z(H)\subseteq cX_{\tilde{r_{p}}}.$ Now let c∉z(H). There is some E⊆H such that $\mathrm{cvE}\subseteq {\left(\mathrm{cX}\right)}_{p}=cX_{p}$ and *E_r_* = *H*. Consequently, $\mathrm{vE}\subseteq X_{p},$ and thus $v\in X_{\tilde{r_{p}}}.$ We infer that $z\in cX_{\tilde{r_{p}}}.$
  Putting all these parts together shows that $\tilde{r_{p}}$ is an ideal system on *H*. We infer by Claim 2 that $X_{\tilde{r_{p}}}\subseteq \underset{F\subseteq X,|F|<\infty }{\cup }F_{\tilde{r_{p}}},$ and hence $\tilde{r_{p}}$ is finitary. We have already shown (below the proof of Claim 2) that $X_{p}\subseteq X_{\tilde{r_{p}}}.$ Moreover, $X_{\tilde{r_{p}}}\subseteq X_{r}$ by Claim 2. This implies that $p\leq \tilde{r_{p}}\leq r.$
2. To show that *r*-$\max(H)=\tilde{r_{p}}$-max(H) it is sufficient to show that every $M\in \tilde{r_{p}}$-max(H) is an *r*-ideal of *H*. Let $M\in \tilde{r_{p}}$-max(H). Assume that *M* is not an *r*-ideal of *H*. Then *M_r_* = *H*. We have that $1M\subseteq M_{p},$ and hence $1\in M_{\tilde{r_{p}}}=M,$ a contradiction.
  Now let X⊆H. Let $x\in X_{\tilde{r_{p}}}$ and N∈r-max(H). Then $\mathrm{xE}\subseteq X_{p}$ and *E_r_* = *H* for some E⊆H, and thus there is some y∈E∖N. It follows that $\mathrm{xy}\in X_{p},$ and hence $x\in y^{-1}X_{p}\subseteq {\left(X_{p}\right)}_{N}.$ This implies that $X_{\tilde{r_{p}}}\subseteq {\left(X_{p}\right)}_{N}$ for every N∈r-max(H). Moreover, we have that
  $$\underset{M\in r\text{-}\max(H)}{\cap }{\left(X_{p}\right)}_{M}=\underset{M\in {\tilde{r}}_{p}\text{-}\max(H)}{\cap }{\left(X_{p}\right)}_{M}\subseteq \underset{M\in {\tilde{r}}_{p}\text{-}\max(H)}{\cap }{\left(X_{{\tilde{r}}_{p}}\right)}_{M}=X_{{\tilde{r}}_{p}}.$$
3. Since $\tilde{r_{p}}\leq r$ by (1), we have clearly that $I_{\tilde{r_{p}}}^{*}(H)\subseteq I_{r}^{*}(H).$ Now let $I\in I_{r}^{*}(H).$ Assume that $I\notin I_{\tilde{r_{p}}}^{*}(H).$ Then ${\left(II^{-1}\right)}_{\tilde{r_{p}}}⊊H,$ and hence there is some $M\in \tilde{r_{p}}$-max(H) such that $II^{-1}\subseteq M.$ We infer by (2) that M∈r-max(H), and hence $H={\left(II^{-1}\right)}_{r}\subseteq M_{r}=M,$ a contradiction. It remains to show that the $\tilde{r_{p}}$-multiplication and the *r*-multiplication coincide on $I_{\tilde{r_{p}}}^{*}(H).$ Let $J,L\in I_{\tilde{r_{p}}}^{*}(H).$ Then ${\left(\mathrm{JL}\right)}_{\tilde{r_{p}}}\in I_{\tilde{r_{p}}}^{*}(H)\subseteq I_{r}(H).$ We infer that ${\left(\mathrm{JL}\right)}_{\tilde{r_{p}}}={\left({\left(\mathrm{JL}\right)}_{\tilde{r_{p}}}\right)}_{r}={\left(\mathrm{JL}\right)}_{r},$ since $\tilde{r_{p}}\leq r$ by (1).
4. Let *p* be modular and let *I*, *J*, *N* be $\tilde{r_{p}}$-ideals of *H* such that I⊆N. Let $x\in {\left(I\cup J\right)}_{\tilde{r_{p}}}\cap N.$ Then there is some E⊆H such that $\mathrm{xE}\subseteq {\left(I\cup J\right)}_{p}$ and *E_r_* = *H*, and thus $\mathrm{xE}\subseteq {\left(I\cup J\right)}_{p}\cap N={\left(I\cup (J\cap N)\right)}_{p}.$ We infer that $x\in {\left(I\cup (J\cap N)\right)}_{\tilde{r_{p}}}.$
5. Let *m* and *n* be finitary ideal systems on *H* such that $p\leq m\leq \tilde{r_{p}}\leq n\leq r$ and X⊆H. First let $x\in X_{\tilde{n_{m}}}.$ Then there is some finite E⊆H such that $\mathrm{xE}\subseteq X_{m}$ and *E_n_* = *H*. Then $\mathrm{xE}\subseteq X_{\tilde{r_{p}}}$ and *E_r_* = *H*. As shown in (1), we have that $\mathrm{xEF}\subseteq X_{p}$ for some F⊆H with *F_r_* = *H*. Observe that ${\left(\mathrm{EF}\right)}_{r}=H,$ and thus $x\in X_{\tilde{r_{p}}}.$

Now let $x\in X_{\tilde{r_{p}}}.$ Then $\mathrm{xE}\subseteq X_{p}$ and *E_r_* = *H* for some E⊆H. By (2) we have that $E_{\tilde{r_{p}}}=H.$ Therefore, $\mathrm{xE}\subseteq X_{m}$ and *E_n_* = *H*, and hence $x\in X_{\tilde{n_{m}}}.$ □

If p≤r are finitary ideal systems on *H* and *p* is modular, then we say (in view of Lemma 2.1(4)) that $\tilde{r_{p}}$ is the *p-modularization* of *r*. Set $w_{p}=\tilde{t_{p}}$ and *w* = *w_s_*. We have that *s* is the finest ideal system on *H*, *t* is the coarsest finitary ideal system on *H* and *v* is the coarsest ideal system on *H*. Furthermore, s≤w≤t≤v and if *H* is an integral domain, then $s\leq d\leq w_{d}\leq t\leq v.$ Note that both the *s*-system and the *d*-system are modular finitary ideal systems. In what follows, we use the remarks of this paragraph without further citation.

## 3. Results for finitary ideal systems

Let *r* be a finitary ideal system on *H*. We say that *H* is an *r-SP-monoid* if every *r*-ideal of *H* is a finite *r*-product of radical *r*-ideals of *H*. Moreover, *H* is called *radical factorial* if every principal ideal of *H* is a finite product of radical principal ideals of *H*. Furthermore, *H* is called *factorial* if every principal ideal of *H* is a finite product of prime principal ideals of *H* (equivalently, every nontrivial prime *t*-ideal of *H* contains a nontrivial prime principal ideal of *H*). We say that *H* is a *valuation monoid* if the principal ideals of *H* are pairwise comparable (equivalently, the *s*-ideals of *H* are pairwise comparable). Also note that if *H* is a valuation monoid, then *s* =* r* = *t* (i.e., the *s*-system is the unique finitary ideal system on *H*). Moreover, if H=G, then *H* is called a *discrete valuation monoid* (or a *DVM*) if every *s*-ideal of *H* is principal (equivalently, every prime *s*-ideal of *H* is principal). We say that *H* satisfies the *Principal Ideal Theorem* if for each nontrivial principal ideal *I* of *H* we have that P(I)⊆X(H). Finally, *H* is called *r-local* if $H\setminus H^{\times }$ is an *r*-ideal of *H* (equivalently, |r-max(H)|=1). Observe that if *H* is *r*-local, then $C_{r}(H)$ is trivial.

It is easy to see that if the radical of every nontrivial principal ideal of *H* is *r*-invertible or every nontrivial principal ideal of *H* is a finite *r*-product of radical *r*-ideals of *H* (in particular if *H* is radical factorial or an *r*-SP-monoid), then *H_M_* is radical factorial for each M∈r-max(H). (In the first case we can show that the radical of every principal ideal of *H_M_* is principal for each M∈r-max(H) and then apply [19, Proposition 2.10].) The main purpose of this section is to present new characterizations of *r*-almost Dedekind monoids, *r*-almost Dedekind *r*-SP-monoids and *r*-Bézout *r*-SP-monoids.

Proposition 3.1. *Let r be a finitary ideal system on H such that H_M_ is radical factorial for each* M∈r*-*max(H)*. Then* $\underset{P\in X(H)}{\cap }H_{P}=H$*, H_Q_ is a DVM for each* Q∈X(H) *and* P(I)⊆X(H) *for each r-invertible r-ideal I of H.*

Proof. By [19, Proposition 2.4] we have for each M∈r-max(H) that $H_{M}=\underset{P\in X(H_{M})}{\cap }{\left(H_{M}\right)}_{P},{\left(H_{M}\right)}_{Q}$ is a DVM for each $Q\in X(H_{M})$ and $P(xH_{M})\subseteq X(H_{M})$ for each $x\in H_{M}^{•}.$ It is easy to see that $X(H_{M})=\{P_{M}|P\in X(H),P\subseteq M\}$ for each M∈r-max(H).

We prove that $\underset{P\in X(H)}{\cap }H_{P}=H.$ If M∈r-max(H), then

$$H_{M}=\underset{Q\in X(H_{M})}{\cap }{\left(H_{M}\right)}_{Q}=\underset{P\in X(H),P\subseteq M}{\cap }{\left(H_{M}\right)}_{P_{M}}=\underset{P\in X(H),P\subseteq M}{\cap }H_{P}.$$

It follows that

$$H=\underset{M\in r\text{-}\max(H)}{\cap }H_{M}=\underset{M\in r\text{-}\max(H)}{\cap }\underset{P\in X(H),P\subseteq M}{\cap }H_{P}=\underset{P\in X(H)}{\cap }H_{P}.$$

Let Q∈X(H). Then $Q_{M}\in X(H_{M}),$ and hence $H_{Q}={\left(H_{M}\right)}_{Q_{M}}$ is a DVM.

Finally we show that P(I)⊆X(H) for each *r*-invertible *r*-ideal *I* of *H*. Let *I* be an *r*-invertible *r*-ideal of *H* and P∈P(I). There is some M∈r-max(H) such that P⊆M. Observe that *I_M_* is a nontrivial principal ideal of *H_M_* and $P_{M}\in P(I_{M})\subseteq X(H_{M}).$ Therefore, there is some $P^{\prime }\in X(H)$ such that $P^{\prime }\subseteq M$ and $P_{M}={\left(P^{\prime }\right)}_{M}.$ This implies that $P=P_{M}\cap H={\left(P^{\prime }\right)}_{M}\cap H=P^{\prime }\in X(H).$ □

Let *r* be a finitary ideal system on *H*. The monoid *H* is called *r-treed* if for all M∈r-max(H), it follows that the prime *r*-ideals of *H* that are contained in *M* form a chain. Moreover, *H* is called an *r-almost Dedekind monoid* (or an *almost r-Dedekind monoid* in the terminology of [19]) if *H* =* G* or if *H_M_* is a DVM for each M∈r-max(H).

Lemma 3.2. *Let r be a finitary ideal system on H such that every nontrivial prime r-ideal of H contains an r-invertible radical r-ideal of H*.

1. *If the prime r-ideals of H form a chain and* H=G*, then H is a DVM.*
2. If H is r-treed, then H is an r-almost Dedekind r-SP-monoid.

Proof.

1. Let the prime *r*-ideals of *H* form a chain and let H=G. Then *H* is *r*-local, and thus every *r*-invertible *r*-ideal of *H* is principal. Moreover, every radical *r*-ideal of *H* is a prime *r*-ideal of *H*. Therefore, every nontrivial prime *r*-ideal of *H* contains a nontrivial prime principal ideal of *H*. Let Ω be the set of all elements of *H* which can be represented as a product of a unit of *H* times a (possibly empty) finite product of nonzero prime elements of *H*. Assume that *H* is not factorial. Then there is some nonzero x∈H∖Ω. It is straightforward to show that xH∩Ω=∅. Since Ω is a multiplicatively closed subset of *H*, *xH* is an *r*-ideal of *H* and *r* is finitary, we infer that xH⊆P and P∩Ω=∅ for some prime *r*-ideal *P* of *H*. Since *P* contains a nonzero prime principal ideal of *H*, we have that P∩Ω=∅, a contradiction. This implies that *H* is a factorial monoid. Since the prime *r*-ideals of *H* form a chain, we have that |X(H)|=1, and thus *H* is a DVM.
2. Let *H* be *r*-treed and M∈r-max(H). Clearly, the prime *r_M_*-ideals of *H_M_* form a chain and every nontrivial prime *r_M_*-ideal of *H_M_* contains an *r_M_*-invertible radical *r_M_*-ideal of *H_M_*. Therefore, *H_M_* is a DVM by (1), and hence *H* is an *r*-almost Dedekind monoid. It follows from [19, Corollary 3.4] that *H* is an *r*-SP-monoid. □

Lemma 3.3. *Let r be a finitary ideal system on H,* k∈N,P∈X(H) *and I_i_ a nontrivial radical r-ideal of H for each* i∈[1,k+1] *such that* $\cup_{i=1}^{k+1}I_{i}\subseteq P$*. Then* ${\left(\prod_{i=1}^{k+1}I_{i}\right)}_{r}$ *does not contain the k-th power of any nonzero radical element of H*.

Proof. Suppose to the contrary that there is some nonzero radical element x∈H such that $x^{k}\in {\left(\prod_{i=1}^{k+1}I_{i}\right)}_{r}.$ We infer that x∈P. It follows that $P_{P}=xH_{P}={\left(I_{j}\right)}_{P}$ for each j∈[1,k+1], and hence $P_{P}^{k}=x^{k}H_{P}\subseteq {\left({\left(\prod_{j=1}^{k+1}I_{j}\right)}_{r}\right)}_{P}={\left(\prod_{j=1}^{k+1}{\left(I_{j}\right)}_{P}\right)}_{r_{P}}={\left(x^{k+1}H_{P}\right)}_{r_{P}}=x^{k+1}H_{P}.$ Therefore, $x^{k}H_{P}=x^{k+1}H_{P},$ and hence $x\in H_{P}^{\times },$ a contradiction. □

Proposition 3.4. *Let r be a finitary ideal system on H and let the radical of every principal ideal of H be principal.*

1. *For each nontrivial r-finitely generated r-ideal I of H there is some nonzero* z∈H *such that* {P∈X(H)|I⊆P}={P∈X(H)|z∈P}.
2. $C_{r}(H)$ *is trivial*.

Proof.

1. Claim 1. If *a*, *b* are nonzero radical elements of *H* such that *b* divides *a*, then
  $$\left\{P\in X(H)|\frac{a}{b}\in P\right\}=\{P\in X(H)|a\in P,b\notin P\}.$$
  To prove Claim 1 let a,b∈H be nonzero radical elements of *H* such that *b* divides *a*. First let P∈X(H) be such that $\frac{a}{b}\in P.$ It is obvious that a∈P. Since *aH_P_* is a nonzero radical ideal of *H_P_* we have that *aH_P_* = *P_P_*. Suppose that b∈P. Then $a\in P^{2},$ and hence $aH_{P}=P_{P}=P_{P}^{2}=a^{2}H_{P}.$ Therefore, $a\in H_{P}^{\times },$ a contradiction. We infer that b∉P. The converse inclusion is trivially satisfied. □(Claim 1)
  Claim 2. For all nonzero x,y∈H there is some nonzero z∈H such that $\left\{P\in X(H)|{\left(\mathrm{xH}\cup \mathrm{yH}\right)}_{r}\subseteq P\}=\{P\in X(H)|z\in P\right\}.$
  To prove Claim 2 let x,y∈H be nonzero. There exist nonzero radical elements a,b,c∈H such that $\sqrt{\mathrm{xyH}}=\mathrm{aH},\sqrt{\mathrm{xH}}=\mathrm{bH}$ and $\sqrt{\mathrm{yH}}=\mathrm{cH}.$ We have that aH=bH∩cH. Moreover, $\sqrt{\frac{a}{b}H}\cap \sqrt{\frac{a}{c}H}=\sqrt{\frac{a^{2}}{\mathrm{bc}}H}=\mathrm{dH}$ for some nonzero radical element d∈H. Set $z=\frac{a}{d}.$
  It follows by Claim 1 that $\{P\in X(H)|d\in P\}=\{P\in X(H)|\frac{a}{b}\in P\}\cup \{P\in X(H)|\frac{a}{c}\in P\}=\{P\in X(H)|a\in P,(b\notin P$ or c∉P)}. We infer by Claim 1 that {P∈X(H)|x,y∈P}={P∈X(H)|b,c∈P}={P∈X(H)|a∈P,d∉P}={P∈X(H)|z∈P}. □(Claim 2) The statement now follows by induction from Claim 2.
2. Claim. The radical of every *r*-invertible *r*-ideal of *H* is principal.

To prove the claim let *I* be an *r*-invertible *r*-ideal of *H*. By Proposition 3.1, we have that P(I)⊆X(H). It follows by (1) that there is some nonzero z∈H such that P(I)={P∈X(H)|I⊆P}={P∈X(H)|z∈P}=P(zH), and hence $\sqrt{I}=\sqrt{\mathrm{zH}}$ is a principal ideal of *H*. □(Claim)

Now let *J* be an *r*-invertible *r*-ideal of *H*. By the claim there is some nonzero radical $z_{1}\in H$ such that $\sqrt{J}=z_{1}H.$ Therefore, $z_{1}^{k}\in J$ for some k∈N.

Next we recursively construct nonzero radical elements *z_i_* of *H* such that $z_{i}H=\sqrt{{\left(\prod_{j=1}^{i-1}z_{j}\right)}^{-1}J}$ for each i∈[1,k+1]. Note that $z_{1}H=\sqrt{{\left(\prod_{j=1}^{1-1}z_{j}\right)}^{-1}J}.$ Now let i∈[1,k] and suppose that we have already constructed the first *i* elements. It follows that ${\left(\prod_{j=1}^{i-1}z_{j}\right)}^{-1}J\subseteq z_{i}H,$ and thus ${\left(\prod_{j=1}^{i}z_{j}\right)}^{-1}J\subseteq H.$ Set $L={\left(\prod_{j=1}^{i}z_{j}\right)}^{-1}J.$ Then $(\prod_{j=1}^{i}z_{j})L_{r}={\left((\prod_{j=1}^{i}z_{j})L\right)}_{r}=J_{r}=J,$ and hence *L* is an *r*-ideal of *H*. Since $J={\left(L\prod_{j=1}^{i}z_{j}H\right)}_{r}$ and *J* is *r*-invertible, we infer that *L* is *r*-invertible. By the claim there is some nonzero radical $z_{i+1}\in H$ such that $\sqrt{L}=z_{i+1}H.$ This completes the construction.

Assume that $z_{k+1}\notin H^{\times }.$ Then there is some $P\in P(z_{k+1}H).$ It follows from Proposition 3.1 that P∈X(H). Observe that $z_{i}H\subseteq z_{i+1}H$ for each i∈[1,k], and thus $\cup_{i=1}^{k+1}z_{i}H\subseteq P.$ Moreover, we have that $z_{1}^{k}\in J\subseteq \prod_{j=1}^{k+1}z_{j}H={\left(\prod_{j=1}^{k+1}z_{j}H\right)}_{r},$ which contradicts Lemma 3.3. Therefore, $z_{k+1}\in H^{\times },$ and hence $\sqrt{{\left(\prod_{j=1}^{k}z_{j}\right)}^{-1}J}=z_{k+1}H=H.$ This implies that ${\left(\prod_{j=1}^{k}z_{j}\right)}^{-1}J=H.$ Consequently, $J=(\prod_{j=1}^{k}z_{j})H$ is a principal ideal of *H*. □

Proposition 3.5. *Let* H=G *and r a finitary ideal system on H and let H be r-local such that the radical of every r-finitely generated r-ideal of H is principal. Then H is a DVM.*

Proof. By Proposition 3.1, *H* satisfies the Principal Ideal Theorem. Assume that *H* is not a valuation monoid. Then there exist x,y∈H such that xH⊈yH and yH⊈xH. Using the fact that the radical of every *r*-finitely generated *r*-ideal of *H* is principal, we can recursively construct nonzero radical elements *z_i_* of *H* such that for every i∈N,

$$\prod_{j=1}^{i-1}z_{j}|x\text{, }\prod_{j=1}^{i-1}z_{j}|y\text{ and }z_{i}H=\sqrt{{\left(\frac{x}{\prod_{j=1}^{i-1}z_{j}}H\cup \frac{y}{\prod_{j=1}^{i-1}z_{j}}H\right)}_{r}}.$$

For i∈N set $w_{i}=\frac{x}{\prod_{j=1}^{i-1}z_{j}}$ and $v_{i}=\frac{y}{\prod_{j=1}^{i-1}z_{j}}.$ Observe that if i∈N, then *w_i_H* and *v_i_H* are not comparable, and hence $w_{i},v_{i}\in H\setminus H^{\times }.$ Since *H* is *r*-local, we infer that $z_{i}\in H\setminus H^{\times }$ for all i∈N. There is some k∈N such that $z_{1}^{k}\in {\left(\mathrm{xH}\cup \mathrm{yH}\right)}_{r}=(\prod_{i=1}^{k}z_{i}){\left(w_{k+1}H\cup v_{k+1}H\right)}_{r}\subseteq {\left(\prod_{i=1}^{k+1}z_{i}H\right)}_{r}.$ Also note that $\cup_{i=1}^{k+1}z_{i}H=z_{k+1}H\subseteq P$ for some P∈X(H), which contradicts Lemma 3.3.

Consequently, *H* is a valuation monoid. It follows by Lemma 3.2(1) that *H* is a DVM. □

Let *r* be a finitary ideal system on *H*. We say that *H* satisfies the *r-prime power condition* if every primary *r*-ideal of *H* is an *r*-power of its radical. Note that every *r*-SP-monoid satisfies the *r*-prime power condition (see [19, Proposition 3.10(1)]). Moreover, *H* satisfies the *strong r-prime power condition* if every *r*-ideal of *H* with prime radical is an *r*-power of its radical. Finally, *H* is called *primary r-ideal inclusive* if for all P,Q∈r-spec(H) such that P⊊Q it follows that $P\subseteq I⊊\sqrt{I}\subseteq Q$ for some primary *r*-ideal *I* of *H*. Now let *I* be an *r*-ideal of *H*. We say that *I* is *r-cancellative* if for all *r*-ideals *J* and *L* of *H* such that ${\left(\mathrm{IJ}\right)}_{r}={\left(\mathrm{IL}\right)}_{r}$ it follows that *J* =* L*. Moreover, *I* is called *r-half cancellative* (or *r-unit-cancellative*) if for all $J\in I_{r}(H)$ with $I={\left(\mathrm{IJ}\right)}_{r}$ it follows that *J* =* H*.

Let $T\subseteq H^{•}$ a multiplicatively closed subset. Note that if *H* satisfies the (strong) *r*-prime power condition, then *T*^−1^*H* satisfies the (strong) *T*^−1^*r*-prime power condition. Moreover, if *H* is primary *r*-ideal inclusive, then *T*^−1^*H* is primary *T*^−1^*r*-ideal inclusive. (By [19, Lemma 3.8] it remains to show that if *H* satisfies the strong *r*-prime power condition, then *T*^−1^*H* satisfies the strong *T*^−1^*r*-prime power condition. Let *H* satisfy the strong *r*-prime power condition and let *J* be a *T*^−1^*r*-ideal of *T*^−1^*H* with prime radical. Set I=J∩H. Then *I* is an *r*-ideal of *H* and $J=T^{-1}I.$ Since $\sqrt{J}$ is a prime *T*^−1^*r*-ideal of *T*^−1^*H*, we have that $\sqrt{I}=\sqrt{J}\cap H$ is a prime *r*-ideal of *H*. Observe that $T^{-1}\sqrt{I}=\sqrt{T^{-1}I}=\sqrt{J}.$ Therefore, $I={\left({\left(\sqrt{I}\right)}^{k}\right)}_{r}$ for some k∈N, and thus $J=T^{-1}I={\left({\left(\sqrt{J}\right)}^{k}\right)}_{T^{-1}r}.$) In what follows, we use the remarks of this paragraph without further citation.

Proposition 3.6. [cf. [6, Theorem 2.14 and Proposition 2.16], [5, Theorem 1.1] and [14, Theorems 4.5 and 4.6]] *Let* H=G *and r a finitary ideal system on H. The following are equivalent*:

1. H is an r-almost Dedekind monoid.
2. H satisfies the strong r-prime power condition and every nontrivial r-ideal of H is r-cancellative.
3. *For all nonzero* x∈H *and* P∈P(xH)*, P is r-half cancellative and every r-ideal of H whose radical is P is an r-power of its radical.*
4. H is r-treed and satisfies the strong r-prime power condition.
5. H satisfies the strong r-prime power condition and r is modular.
6. *r-*max(H)=X(H) *and H satisfies the r-prime power condition.*
7. H satisfies the r-prime power condition and the Principal Ideal Theorem, and H is primary r-ideal inclusive.

Proof. Claim 1. If *H* satisfies the *r*-prime power condition and P∈P(xH) for some nonzero x∈H, then *P_P_* is principal.

Let x∈H be nonzero and P∈P(xH). Since *P_P_* is the only prime *s*-ideal of *H_P_* such that $x\in P_{P},$ we infer that $\sqrt{xH_{P}}=P_{P},$ Note that *H_P_* satisfies the *r_P_*-prime power condition (by the discussion above), and thus $xH_{P}={\left(P_{P}^{k}\right)}_{r_{P}}$ for some k∈N. (Note that $P_{P}\in r_{P}$-$\max(H_{P}),$ and thus *xH_P_* is *P_P_*-primary.) Therefore, *P_P_* is *r_P_*-invertible, and hence *P_P_* is principal, since *H_P_* is *r_P_*-local. □(Claim 1)

(1) ⇒ (2),(5): Clearly, *r*-max(H)=X(H). Let *I* be a nontrivial *r*-ideal of *H* and *J*, *L r*-ideals of *H* such that ${\left(\mathrm{IJ}\right)}_{r}={\left(\mathrm{IL}\right)}_{r}.$ If M∈r-max(H), then *I_M_* = *xH_M_* for some nonzero $x\in H_{M}$ and hence $xJ_{M}=I_{M}J_{M}={\left(I_{M}J_{M}\right)}_{r_{M}}={\left({\left(\mathrm{IJ}\right)}_{r}\right)}_{M}={\left({\left(\mathrm{IL}\right)}_{r}\right)}_{M}={\left(I_{M}L_{M}\right)}_{r_{M}}=I_{M}L_{M}=xJ_{M}.$ We infer that *J_M_* = *L_M_* for each M∈r-max(H), and thus *J* =* L*.

Now let *I* be a nontrivial *r*-ideal of *H* with prime radical. Set $M=\sqrt{I}.$ Observe that *H_M_* is a DVM, and thus every nontrivial *s*-ideal of *H_M_* is a power of *M_M_*. Consequently, $I_{M}=M_{M}^{k}={\left(M_{M}^{k}\right)}_{r_{M}}={\left({\left(M^{k}\right)}_{r}\right)}_{M}$ for some k∈N. Since M∈r-max(H), both *I* and ${\left(M^{k}\right)}_{r}$ are *M*-primary *r*-ideals of *H*, and hence $I=I_{M}\cap H={\left({\left(M^{k}\right)}_{r}\right)}_{M}\cap H={\left(M^{k}\right)}_{r}.$ It is clear that *r* is modular.

(2) ⇒ (3): This is obvious.

(3) ⇒ (4): It is sufficient to show that every *r*-maximal *r*-ideal of *H* is of height one. Let *M* be an *r*-maximal *r*-ideal of *H*. Assume that *M* is not of height one, then there exist x∈M∖{0} and P∈P(xH) such that P⊊M. By Claim 1 there is some $y\in P_{P}$ such that *P_P_* = *yH_P_*. We have that $\sqrt{{\left(\mathrm{PM}\right)}_{r}}=\sqrt{P}\cap \sqrt{M}=P,$ and thus ${\left(\mathrm{PM}\right)}_{r}={\left(P^{k}\right)}_{r}$ for some k∈N. Since ${\left(P^{2}\right)}_{r}\subseteq {\left(\mathrm{PM}\right)}_{r}\subseteq P$ and *P* is *r*-half cancellative, we infer that ${\left(\mathrm{PM}\right)}_{r}={\left(P^{2}\right)}_{r},$ and thus $yH_{P}={\left(P_{P}M_{P}\right)}_{r_{P}}={\left(P_{P}^{2}\right)}_{r_{P}}=y^{2}H_{P}.$ This implies that $P_{P}=yH_{P}=H_{P},$ a contradiction.

(4) ⇒ (6): It is sufficient to show that every *r*-maximal *r*-ideal of *H* is of height one. Let *M* be an *r*-maximal *r*-ideal of *H*. First we show that the radical of every nontrivial principal ideal of *H_M_* is *r_M_*-invertible. Let *I* be a nontrivial proper principal ideal of *H_M_*. The prime *r_M_*-ideals of *H_M_* form a chain, and hence $\sqrt{I}$ is a prime *r_M_*-ideal of *H_M_*. Since *H_M_* satisfies the strong *r_M_*-prime power condition, we have that $I=((\sqrt[{H_{M}}]{I}{)}^{k}{)}_{r}{}_{M}$ for some k∈N. This implies that $\sqrt[{H_{M}}]{I}$ is *r_M_*-invertible.

We infer that every nontrivial prime *r_M_*-ideal of *H_M_* contains an *r_M_*-invertible radical *r_M_*-ideal of *H_M_*. It follows by Lemma 3.2(1) that *H_M_* is a DVM, and hence M∈X(H).

(5) ⇒ (6): Assume that *r*-max(H)=X(H). Then there exist $y\in H^{•},P\in P(\mathrm{yH})$ and M∈r-max(H) such that P⊊M. By Claim 1 there is some x∈P such that *P_P_* = *xH_P_*. Observe that $\sqrt{{\left({\left(P^{2}\right)}_{r}\cup \mathrm{xM}\right)}_{r}}=P,$ and hence ${\left({\left(P^{2}\right)}_{r}\cup \mathrm{xM}\right)}_{r}={\left(P^{k}\right)}_{r}$ for some k∈N. If k≥2, then $\mathrm{xM}\subseteq {\left(P^{2}\right)}_{r},$ and thus $xH_{P}=xM_{P}\subseteq {\left(P_{P}^{2}\right)}_{r_{P}}=x^{2}H_{P},$ a contradiction. Therefore, ${\left({\left(P^{2}\right)}_{r}\cup \mathrm{xM}\right)}_{r}=P.$ If z∈H is such that $\mathrm{xz}\in {\left(P^{2}\right)}_{r},$ then $\mathrm{xz}\in {\left({\left(P^{2}\right)}_{r}\right)}_{P}=x^{2}H_{P},$ and thus $z\in xH_{P}\cap H=P.$ We infer that $x\in P\cap \mathrm{xH}={\left(\mathrm{xM}\cup {\left(P^{2}\right)}_{r}\right)}_{r}\cap \mathrm{xH}={\left(\mathrm{xM}\cup ({\left(P^{2}\right)}_{r}\cap \mathrm{xH})\right)}_{r}\subseteq {\left(\mathrm{xM}\cup \mathrm{xP}\right)}_{r}=\mathrm{xM},$ a contradiction.

(6) ⇒ (7): It is clear that *H* satisfies the Principal Ideal Theorem. It follows from [19, Proposition 3.9] that *H* is primary *r*-ideal inclusive.

(7) ⇒ (1): Recall that a prime *r*-ideal *P* of *H* is called *r*-branched if there exists a *P*-primary *r*-ideal *I* of *H* with I=P.

Claim 2. For each *r*-branched prime *r*-ideal *P* of *H*, we have that P∈X(H) and *H_P_* is a DVM.

Let *P* be an *r*-branched prime *r*-ideal of *H*. Then *P_P_* is a principal ideal of *H_P_* by [19, Proposition 5.2(1)]. There is some $x\in H^{•}$ such that *P_P_* = *xH_P_*. Observe that P∈P(xH)⊆X(H), and hence $P_{P}\in X(H_{P}).$ Therefore, every prime *s*-ideal of *H_P_* is principal, and hence *H_P_* is a DVM. □(Claim 2)

Let M∈r-max(H). It is sufficient to show that M∈X(H) (then *M* is *r*-branched, and hence *H_M_* is a DVM by Claim 2). Assume that M∉X(H). Then there is some nontrivial prime *r*-ideal *P* of *H* such that P⊊M. Since *H* is primary *r*-ideal inclusive, we can find an *r*-branched prime *r*-ideal *Q* of *H* such that P⊊Q. By Claim 2 we have that Q∈X(H), a contradiction. □

Lemma 3.7. *Let r be a modular finitary ideal system on H. Then H is primary r-ideal inclusive*.

Proof. Let P,Q∈r-spec(H) be such that P⊊Q. There exist x∈Q∖P and $L\in P({\left(P\cup x^{2}H\right)}_{r})$ such that L⊆Q. Set $I={\left({\left(P\cup x^{2}H\right)}_{r}\right)}_{L}\cap H.$ Observe that *I* is an *L*-primary *r*-ideal of *H*. It remains to show that I=L. Assume to the contrary that *I* =* L*. Then $x\in {\left({\left(P\cup x^{2}H\right)}_{r}\right)}_{L}={\left(P_{L}\cup x^{2}H_{L}\right)}_{r_{L}}.$ Since *r_L_* is a modular finitary ideal system on *H_L_*, we obtain that $xH_{L}={\left(x^{2}H_{L}\cup P_{L}\right)}_{r_{L}}\cap xH_{L}={\left(x^{2}H_{L}\cup (P_{L}\cap xH_{L})\right)}_{r_{L}}={\left(x^{2}H_{L}\cup xP_{L}\right)}_{r_{L}}=x{\left(xH_{L}\cup P_{L}\right)}_{r_{L}}\subseteq xL_{L}.$ Therefore, $H_{L}\subseteq L_{L},$ a contradiction. □

Let *r* be a finitary ideal system on *H*. Then *H* is called an *r-Prüfer monoid*, resp. an *r-Bézout monoid*, if every nontrivial *r*-finitely generated *r*-ideal of *H* is *r*-invertible, resp. principal. Note that *H* is an *r*-Bézout monoid if and only if *H* is an *r*-Prüfer monoid and $C_{r}(H)$ is trivial. Note that *H* is an *r*-Prüfer monoid if and only if *H_M_* is a valuation monoid for all M∈r-max(H). In particular, if *H* is an *r*-Prüfer monoid, then *H* is *r*-treed and *r* is modular. Moreover, *H* is an *s*-Prüfer monoid if and only if *H* is a valuation monoid.

Corollary 3.8. [cf. [5, 10]] *Let* H=G *and let p and r be finitary ideal systems on H such that p is modular and* p≤r*. The following are equivalent:*

1. H is an r-almost Dedekind monoid.
2. *H is an* $\tilde{r_{p}}$*-almost Dedekind monoid.*
3. $\tilde{r_{p}}$*-*max(H)=X(H) *and H satisfies the* $\tilde{r_{p}}$*-prime power condition.*
4. *H satisfies the strong* $\tilde{r_{p}}$*-prime power condition.*
5. *H satisfies the* $\tilde{r_{p}}$*-prime power condition and the Principal Ideal Theorem.*

If these equivalent conditions are satisfied, then $\tilde{r_{p}}=r=t.$

Proof. (1) ⇔ (2): This is an immediate consequence of Lemma 2.1(2).

(2) ⇔ (3) ⇔ (4) ⇔ (5): This follows from Proposition 3.6 and Lemmas 2.1(4) and 3.7.

Now let the equivalent conditions be satisfied. Since $\tilde{r_{p}}\leq r\leq t,$ it is sufficient to show that every $\tilde{r_{p}}$-ideal of *H* is a *t*-ideal of *H*. Let $I\in I_{\tilde{r_{p}}}(H).$ Observe that *H* is an $\tilde{r_{p}}$-Prüfer monoid, and hence every $\tilde{r_{p}}$-finitely generated $\tilde{r_{p}}$-ideal of *H* is a *t*-ideal of *H*. Since $\tilde{r_{p}}$ is finitary, we infer that *I* is a directed union of *t*-ideals of *H*, and hence *I* is a *t*-ideal of *H*. □

Theorem 3.9. *Let r be a finitary ideal system on H. The following are equivalent:*

1. H is an r-almost Dedekind r-SP-monoid.
2. H is r-treed and every nontrivial prime r-ideal of H contains an r-invertible radical r-ideal of H.
3. H satisfies the r-prime power condition, H is primary r-ideal inclusive and each nontrivial prime r-ideal of H contains an r-invertible radical r-ideal.
4. The radical of every nontrivial r-finitely generated r-ideal of H is r-invertible.
5. P(I)⊆X(H) *for every nontrivial r-finitely generated r-ideal I of H and the radical of every nontrivial principal ideal of H is r-invertible.*

Proof. Without restriction let H=G. (1) ⇒ (2), (3): This follows from [19, Corollary 3.4 and Propositions 3.9 and 3.10(1)].

(2) ⇒ (1): This follows from Lemma 3.2(2).

(3) ⇒ (1): First we show that *H* satisfies the Principal Ideal Theorem. Let $x\in H^{•}$ and P∈P(xH). It follows by Claim 1 in the proof of Proposition 3.6 that *P_P_* is principal. Observe that every nontrivial prime *r_P_*-ideal of *H_P_* contains a nontrivial radical principal ideal of *H_P_*, and thus $P_{P}\in X(H_{P})$ by [19, Lemma 2.3(2)] (since *P_P_* is principal and thus minimal above a nontrivial radical principal ideal of *H_P_*). Therefore, P∈X(H).

Consequently, *H* is an *r*-almost Dedekind monoid by Proposition 3.6. By [19, Corollary 3.4] we have that *H* is an *r*-SP-monoid.

(1) ⇒ (4): This follows from [19, Corollary 3.4].

(4) ⇒ (5): Let *I* be a nontrivial *r*-finitely generated *r*-ideal of *H*. We infer by Proposition 3.1 that $P(I)=P(\sqrt{I})\subseteq X(H).$

(5) ⇒ (1): Let M∈r-max(H). Then *H_M_* is *r_M_*-local, *r_M_* is a finitary ideal system on *H_M_* and the radical of every principal of *H_M_* is principal. Let *I* be a nontrivial *r_M_*-finitely generated *r_M_*-ideal of *H_M_*. Then $P(I)\subseteq X(H_{M}).$ (Note that there is some nontrivial *r*-finitely generated *r*-ideal *J* of *H* such that *I* = *J_M_*. Moreover, the contraction of every element of P(I) to *H* is an element of P(J).) By Proposition 3.4(1), there is some nonzero $z\in H_{M}$ such that $P(I)=\{P\in X(H_{M})|I\subseteq P\}=\{P\in X(H_{M})|z\in P\}=P(zH_{M}).$ This implies that $\sqrt{I}=\sqrt{zH_{M}}$ is principal. It follows by Proposition 3.5 that *H_M_* is a DVM. We infer that *H* is an *r*-almost Dedekind monoid. It follows by [19, Corollary 3.4] that *H* is an *r*-SP-monoid. □

Theorem 3.10. *Let r be a finitary ideal system on H. The following are equivalent:*

1. H is an r-Bézout r-SP-monoid.
2. H is a radical factorial r-Bézout monoid.
3. H is r-treed, $C_{r}(H)$ is trivial and every nontrivial prime r-ideal of H contains a nontrivial radical principal ideal of H.
4. H satisfies the r-prime power condition, H is primary r-ideal inclusive and the radical of every principal ideal of H is principal.
5. H is r-treed and the radical of every principal ideal of H is principal.
6. P(I)⊆X(H) for every nontrivial r-finitely generated r-ideal I of H and the radical of every principal ideal of H is principal.
7. The radical of every r-finitely generated r-ideal of H is principal.

Proof. (1) ⇒ (2): Clearly, $C_{r}(H)$ is trivial, and thus *H* is radical factorial by [19, Proposition 3.10(2)].

(2) ⇒ (3): Since *H* is an *r*-Bézout monoid, it is clear that *H* is *r*-treed and $C_{r}(H)$ is trivial. Since *H* is radical factorial, every nontrivial prime *r*-ideal of *H* contains a nontrivial radical principal ideal of *H*.

(3) ⇒ (1): This is an immediate consequence of Theorem 3.9, since every *r*-almost Dedekind monoid with trivial *r*-class group is an *r*-Bézout monoid.

(1) ⇒ (4): It follows from Theorem 3.9 that *H* satisfies the *r*-prime power condition, that *H* is primary *r*-ideal inclusive and that the radical of every nontrivial principal ideal of *H* is *r*-invertible. Since *H* is an *r*-Bézout monoid, we infer that the radical of every principal ideal of *H* is principal.

(4) ⇒ (5): This is an immediate consequence of Theorem 3.9.

(5) ⇒ (6): Without restriction let H=G. It follows by Lemma 3.2(2) and [19, Proposition 2.10] that *H* is an *r*-almost Dedekind *r*-SP-monoid, and hence *r*-max(H)=X(H). Obviously, P(I)⊆X(H) for every nontrivial *r*-finitely generated *r*-ideal *I* of *H*.

(6) ⇒ (7): Let *I* be a nontrivial *r*-finitely generated *r*-ideal of *H*. By Proposition 3.4(1), we have that P(I)={P∈X(H)|I⊆P}={P∈X(H)|z∈P}=P(zH) for some nonzero z∈H. Consequently, $\sqrt{I}=\sqrt{\mathrm{zH}}$ is principal.

(7) ⇒ (1): By Theorem 3.9, *H* is an *r*-almost Dedekind *r*-SP-monoid. We infer by Proposition 3.4(2) that *H* is an *r*-Bézout monoid. □

Next we rediscover several well-known characterizations for (Bézout) SP-domains and we also present some new characterizations.

Corollary 3.11. [cf. [13, Lemma 4.2 and Theorem 4.3] and [18, Corollary 7.7]] *Let R be an integral domain.*

- The following are equivalent*:*
  1. R is an SP-domain.
  2. R is treed and every nonzero prime ideal of R contains an invertible radical ideal of R.
  3. Every primary ideal of R is a power of its radical and every nonzero prime ideal of R contains an invertible radical ideal of R.
  4. Every minimal prime ideal of each nonzero finitely generated ideal of R is of height one and the radical of every nonzero principal ideal of R is invertible.
  5. The radical of every nonzero finitely generated ideal of R is invertible.
- The following are equivalent:
  1. R is a Bézout SP-domain.
  2. R is a radical factorial Bézout domain.
  3. R is treed and the radical of every principal ideal of R is principal.
  4. Every primary ideal of R is a power of its radical and the radical of every principal ideal of R is principal.
  5. Every minimal prime ideal of each nonzero finitely generated ideal of R is of height one and the radical of every principal ideal of R is principal.
  6. The radical of every finitely generated ideal of R is principal.

Proof. This is an easy consequence of Lemma 3.7 and Theorems 3.9 and 3.10. □

Note that there are examples of *t*-SP-monoids that fail to be *t*-almost Dedekind monoids. As shown in [20, Example 4.2] there is some *t*-local *t*-SP-monoid *H* such that every nontrivial *t*-ideal of *H* is *t*-cancellative and *t*-dim(H)=2. In particular, *H* satisfies the *t*-prime power condition and P(I)⊆X(H) for each nontrivial *t*-finitely generated *t*-ideal of *H*. Note that *H* does not satisfy the strong *t*-prime power condition, *H* is not *t*-treed and *H* is not primary *t*-ideal inclusive.

## 4 On the *t*-system and the *w*-system

In this section, we study the *t*-system and its modularizations. We present stronger characterizations for these types of finitary ideal systems than in the section before. Besides that, we investigate the connections with the modularizations $\tilde{r_{p}}$ of a finitary ideal system *r* in general and describe $\tilde{r_{p}}$-SP-monoids and $\tilde{r_{p}}$-Bézout $\tilde{r_{p}}$-SP-monoids. We also show that the *t*-class group of every radical factorial BF-monoid is torsionfree. Let *r* be a finitary ideal system on *H*. We say that *H* is an *r-finite conductor monoid* if xH∩yH is *r*-finitely generated for all x,y∈H.

Proposition 4.1. [cf. [11,24]] *Let* P *be a set of prime s-ideals of H such that* $\underset{P\in P}{\cap }H_{P}=H$ *and H_Q_ is a valuation monoid for every* Q∈P*. Let I and J be t-ideals of H.*

1. *If I, J and* I∩J *are t-finitely generated, then* ${\left(\mathrm{IJ}\right)}_{t}={\left((I\cap J){\left(I\cup J\right)}_{t}\right)}_{t}.$
2. *If I and J are t-invertible and* I∩J *is t-finitely generated, then* I∩J *and* ${\left(I\cup J\right)}_{t}$ *are t-invertible.*
3. If H is a t-finite conductor monoid, then H is a t-Prüfer monoid.

Proof. Observe that r:P(H)→P(H) defined by $X_{r}=\underset{P\in P}{\cap }{\left(X_{s}\right)}_{P}$ for each X⊆H is an ideal system on *H*. This implies that r≤v, and hence $I=\underset{P\in P}{\cap }I_{P}$ for each divisorial ideal *I* of *H*.

1. Let *I*, *J* and I∩J be *t*-finitely generated. Then ${\left(\mathrm{IJ}\right)}_{t}$ and ${\left(I\cup J\right)}_{t}$ are *t*-finitely generated. This implies that ${\left((I\cap J)(I\cup J)\right)}_{t}={\left((I\cap J){\left(I\cup J\right)}_{t}\right)}_{t}$ is *t*-finitely generated. Therefore, it is sufficient to show that ${\left({\left((I\cap J)(I\cup J)\right)}_{t}\right)}_{P}={\left({\left(\mathrm{IJ}\right)}_{t}\right)}_{P}$ for each P∈P. Let P∈P. Since *H_P_* is a valuation monoid, we have that $I_{P}\subseteq J_{P}$ or $J_{P}\subseteq I_{P}.$ Consequently, ${\left({\left((I\cap J)(I\cup J)\right)}_{t}\right)}_{P}={\left((I_{P}\cap J_{P})(I_{P}\cup J_{P})\right)}_{t_{P}}={\left(I_{P}J_{P}\right)}_{t_{P}}={\left({\left(\mathrm{IJ}\right)}_{t}\right)}_{P}.$
2. Let *I* and *J* be *t*-invertible and let I∩J be *t*-finitely generated. Clearly, *I* and *J* are *t*-finitely generated, and thus ${\left((I\cap J){\left(I\cup J\right)}_{t}\right)}_{t}={\left(\mathrm{IJ}\right)}_{t}.$ Since ${\left(\mathrm{IJ}\right)}_{t}$ is t-invertible, we have that ${\left((I\cap J){\left(I\cup J\right)}_{t}\right)}_{t}$ is *t*-invertible, and hence I∩J and ${\left(I\cup J\right)}_{t}$ are *t*-invertible.
3. Let *H* be a *t*-finite conductor monoid. First we show that for each nonempty finite A⊆H and each x∈H it follows that $A_{t}\cap \mathrm{xH}$ is *t*-finitely generated. Let A⊆H be finite and nonempty and x∈H. Let P∈P. Since *H_P_* is a valuation monoid, we have that ${\left(A_{t}\right)}_{P}=AH_{P}.$ We infer that ${\left(A_{t}\cap \mathrm{xH}\right)}_{P}=AH_{P}\cap xH_{P}=\underset{b\in A}{\cup }(bH_{P}\cap xH_{P})=(\underset{b\in A}{\cup }(bH_{P}\cap xH_{P}){)}_{t_{P}}=((\underset{b\in A}{\cup }(\mathrm{bH}\cap \mathrm{xH}){)}_{t}{)}_{P}.$ This implies that $A_{t}\cap \mathrm{xH}=(\underset{b\in A}{\cup }(bH\cap \mathrm{xH}){)}_{t}$ is *t*-finitely generated. Next we show by induction that for each n∈N and all $E\subseteq H^{•}$ with |E|=n it follows that *E_t_* is *t*-invertible. The statement is clearly true for *n* = 1. Now let n∈N and $F\subseteq H^{•}$ be such that |F|=n+1. There exist E⊆F and x∈F∖E such that F=E∪{x} and |E|=n. It follows by the previous claim that $E_{t}\cap \mathrm{xH}$ is *t*-finitely generated. We infer by (2) that $F_{t}={\left(E_{t}\cup \mathrm{xH}\right)}_{t}$ is *t*-invertible. □

Theorem 4.2. *The following are equivalent:*

1. H is a t-almost Dedekind t-SP-monoid.
2. H is a t-finite conductor monoid and every principal ideal of H is a finite t-product of radical t-ideals of H.
3. Every t-ideal of H is a t-product of finitely many pairwise comparable radical t-ideals of H.
4. The radical of every nontrivial principal ideal of H is t-invertible.

Proof. (1) ⇒ (2): This is obvious.

(2) ⇒ (1): By Proposition 3.1 we have that $\underset{P\in X(H)}{\cap }H_{P}=H$ and *H_Q_* is a DVM for every Q∈X(H). It follows by Proposition 4.1(3) that *H* is a *t*-Prüfer monoid, and hence *H* is *t*-treed. Consequently, *H* is a *t*-almost Dedekind *t*-SP-monoid by Theorem 3.9.

(1) ⇒ (3): This follows from [19, Theorem 3.3(2)].

(3) ⇒ (4): Let $x\in H^{•}.$ There exist n∈N and finitely many radical *t*-ideals *I_i_* of *H* such that $I_{i}\subseteq I_{i+1}$ for each i∈[1,n−1] and $\mathrm{xH}={\left(\prod_{i=1}^{n}I_{i}\right)}_{t}.$ This implies that $\sqrt{\mathrm{xH}}=\cap_{i=1}^{n}I_{i}=I_{1}$ is *t*-invertible.

(4) ⇒ (1): By Theorem 3.9 it is sufficient to show that the radical of every nontrivial *t*-finitely generated *t*-ideal of *H* is *t*-invertible.

It follows by Proposition 3.1 that $\underset{P\in X(H)}{\cap }H_{P}=H,$ *H_Q_* is a DVM for each Q∈X(H) and P(A)⊆X(H) for each *t*-invertible *t*-ideal *A* of *H*.

It is sufficient to show by induction that for each n∈N and each $E\subseteq H^{•}$ with |E|=n it follows that $\sqrt{E_{t}}$ is *t*-invertible. The statement is clearly true for *n* = 1. Now let n∈N and $F\subseteq H^{•}$ be such that |F|=n+1. There exist E⊆F and x∈F∖E such that |E|=n and F=E∪{x}. Set $I=\sqrt{E_{t}}$ and $J=\sqrt{\mathrm{xH}}.$ Then *I* and *J* are *t*-invertible radical *t*-ideals of *H*. Observe that $\sqrt{F_{t}}=\sqrt{{\left(I\cup J\right)}_{t}},$ since the radical of every *t*-ideal of *H* is a *t*-ideal of *H*. Moreover, $I\cap J=\sqrt{{\left(\mathrm{xE}\right)}_{t}}$ is *t*-invertible, since |xE|=|E|=n. We infer by Proposition 4.1(2) that ${\left(I\cup J\right)}_{t}$ is *t*-invertible. Note that

(I∪J)t=∩P∈X(H),(I∪J)t⊆PP=(∩P∈X(H),(I∪J)t⊆PPP)∩H=(∩P∈X(H),(I∪J)t⊆PPP)∩(∩P∈X(H),(I∪J)t⊆PHP)=(∩P∈X(H),(I∪J)t⊆P((I∪J)t)P)∩(∩P∈X(H),(I∪J)t⊆P((I∪J)t)P)=∩P∈X(H)((I∪J)t)P=(I∪J)t,

where the first equality holds since $P({\left(I\cup J\right)}_{t})\subseteq X(H),$ and the last equality holds since ${\left(I\cup J\right)}_{t}$ is *t*-finitely generated (and hence divisorial). Therefore, $\sqrt{F_{t}}={\left(I\cup J\right)}_{t}$ is *t*-invertible. □

Theorem 4.3. *Let* H=G *and let p and r be finitary ideal systems on H such that p is modular and* p≤r.

- The following are equivalent:
  1. H is an r-almost Dedekind r-SP-monoid.
  2. *r-*max(H)=t*-*max(H) *and the radical of every nontrivial principal ideal of H is t-invertible.*
  3. *H is an* $\tilde{r_{p}}$*-SP-monoid.*
- The following are equivalent:
  1. H is an r-Bézout r-SP-monoid.
  2. *r-*max(H)=t*-*max(H) *and the radical of every principal ideal of H is principal.*
  3. *H is an* $\tilde{r_{p}}$*-Bézout* $\tilde{r_{p}}$*-SP-monoid*.

Proof. (A) (1) ⇒ (2): First let *H* be an *r*-almost Dedekind *r*-SP-monoid. Clearly, *r*-max(H)=X(H), and since every height-one prime *s*-ideal of *H* is a *t*-ideal, we infer that *r*-max(H)=t-max(H). By Theorem 3.9, the radical of every nontrivial principal ideal of *H* is *r*-invertible. Since r≤t, we have that the radical of every nontrivial principal ideal of *H* is *t*-invertible.

(2) ⇒ (1): Now let *r*-max(H)=t-max(H) and let the radical of every nontrivial principal ideal of *H* be *t*-invertible. It follows by Theorem 4.2 that *H* is a *t*-almost Dedekind monoid, and hence *r*-max(H)=t-max(H)=X(H). Therefore, *H* is *r*-treed and every *t*-invertible *t*-ideal of *H* is an *r*-invertible *r*-ideal of *H*. Consequently, *H* is an *r*-almost Dedekind *r*-SP-monoid by Theorem 3.9.

(2) ⇔ (3): By Lemmas 2.1(4) and 3.7, Theorem 3.9 and [19, Proposition 3.10(1)], we have that *H* is an $\tilde{r_{p}}$-SP-monoid if and only if *H* is an $\tilde{r_{p}}$-almost Dedekind $\tilde{r_{p}}$-SP-monoid. Now applying the equivalence of (1) and (2) to $\tilde{r_{p}}$ and using the fact that *r*-$\max(H)=\tilde{r_{p}}$-max(H) gives us the desired equivalence.

(B) This is an easy consequence of (A), Proposition 3.4(2) and Theorem 3.10. □

Corollary 4.4. *The following are equivalent*:

1. H is a t-almost Dedekind t-SP-monoid.
2. H is a w-SP-monoid.
3. H is a w-finite conductor monoid and every principal ideal of H is a finite w-product of radical w-ideals of H.
4. Every w-ideal of H is a w-product of finitely many pairwise comparable radical w-ideals of H.
5. The radical of every nontrivial principal ideal of H is w-invertible.

Proof. (1) ⇒ (2): By Theorem 4.2, the radical of every nontrivial principal ideal is *t*-invertible. As pointed out before, we have that *w*-max(H)=t-max(H). We infer by Theorem 4.3(A) that *H* is a *w*-almost Dedekind *w*-SP-monoid.

(2) ⇒ (3): This is obvious, since *H* is a *w*-almost Dedekind monoid.

(3) ⇒ (1): Let x,y∈H. Then $\mathrm{xH}\cap \mathrm{yH}=E_{w}$ for some finite E⊆H. Since w≤t, we infer that $\mathrm{xH}\cap \mathrm{yH}={\left(\mathrm{xH}\cap \mathrm{yH}\right)}_{t}={\left(E_{w}\right)}_{t}=E_{t}.$ Therefore, *H* is a *t*-finite conductor monoid. Note that every nontrivial principal ideal of *H* is a finite *w*-product of *w*-invertible radical *w*-ideals of *H*. Therefore, every nontrivial principal ideal of *H* is a finite *t*-product of (*t*-invertible) radical *t*-ideals of *H* by Lemma 2.1(3). The statement now follows from Theorem 4.2.

(2) ⇒ (4) ⇒ (5): This can be proved along the same lines as in Theorem 4.2.

(5) ⇒ (1): Since every *w*-invertible *w*-ideal of *H* is a *t*-invertible *t*-ideal of *H*, the radical of every nontrivial principal ideal of *H* is *t*-invertible. Therefore, *H* is a *t*-almost Dedekind *t*-SP-monoid by Theorem 4.2. □

Corollary 4.5. *The following are equivalent*:

1. H is a t-Bézout t-SP-monoid.
2. H is a w-Bézout w-SP-monoid.
3. The radical of every principal ideal of H is principal.
4. Every principal ideal of H is a product of finitely many pairwise comparable radical principal ideals.

Proof. (1) ⇔ (2) ⇔ (3): This follows from Theorem 4.3(B).

(3) ⇒ (4): It follows by [19, Lemma 2.3(2)] that *H* satisfies the Principal Ideal Theorem. Let x∈H be nonzero. Clearly, there is a sequence ${\left(z_{i}\right)}_{i\in N}$ of nonzero radical elements of *H* such that $\sqrt{(x/\prod_{i=1}^{\ell -1}z_{i})H}=z_{\ell }H$ for each ℓ∈N. Moreover, we have that $z_{\ell }H\subseteq z_{\ell +1}H$ for all ℓ∈N. Since $\sqrt{\mathrm{xH}}=z_{1}H,$ there is some k∈N such that $z_{1}^{k}\in \mathrm{xH}.$ We infer by Lemma 3.3 that $z_{k+1}\in H^{\times },$ and thus $\mathrm{xH}=\prod_{i=1}^{k}z_{i}H.$

(4) ⇒ (3): Let $x\in H^{•}.$ Then there exist n∈N and finitely many radical principal ideals *I_i_* of *H* such that $\mathrm{xH}=\prod_{i=1}^{n}I_{i}$ and $I_{i}\subseteq I_{i+1}$ for all i∈[1,n−1]. It follows that $\sqrt{\mathrm{xH}}=\cap_{i=1}^{n}I_{i}=I_{1}$ is principal. □

Note that *w* can be replaced by *w_p_* in Corollaries 4.4 and 4.5, where *p* is an arbitrary modular finitary ideal system on *H*.

Corollary 4.6. *H is factorial if and only if the radical of every principal ideal of H is principal and H satisfies the ascending chain condition on radical principal ideals*.

Proof. This is an immediate consequence of Theorem 3.10, Corollary 4.5 and [19, Theorem 2.14]. □

Finally, we give a partial positive answer to the (so far) unsolved problem of whether the *t*-class group of a radical factorial monoid is torsionfree. The following result shows that the *t*-class group of a radical factorial monoid has to satisfy a “weak form” of being torsionfree. Let *H* be a monoid and A⊆P(H). A function $\lambda :A\to N_{0}$ is called a length function on A if λ(J)<λ(I) for all I,J∈A with I⊊J. Moreover, *H* is called a *BF-monoid* if the set of nontrivial principal ideals of *H* possesses a length function.

Proposition 4.7. *Let H be a radical factorial monoid,* $k\in N,I\in I_{t}^{*}(H)$ *such that* ${\left(I^{k}\right)}_{t}$ *is principal and* $A=\{{\left(L^{k}\right)}_{t}|L\in I_{t}^{*}(H),I\subseteq L,{\left(L^{k}\right)}_{t}$ *is principal*}.

1. *If* A *possesses a length function, then I is principal.*
2. *If* {P∈X(H)|I⊆P} *is finite, then I is principal.*
3. *If H is a BF-monoid, then* $C_{t}(H)$ *is torsionfree.*

Proof.

1. Let $\lambda :A\to N_{0}$ be a length function on A. It is sufficient to show by induction that for each $n\in N_{0}$ and $L\in I_{t}^{*}(H)$ such that $I\subseteq L,{\left(L^{k}\right)}_{t}$ is principal and $\lambda ({\left(L^{k}\right)}_{t})=n,$ it follows that *L* is principal. Let $n\in N_{0}$ and $L\in I_{t}^{*}(H)$ be such that $I\subseteq L,{\left(L^{k}\right)}_{t}$ is principal and $\lambda ({\left(L^{k}\right)}_{t})=n.$ Without restriction let L=H. There is some radical nonunit x∈H such that $L\subseteq \sqrt{L}=\sqrt{{\left(L^{k}\right)}_{t}}\subseteq \mathrm{xH}.$ Consequently, *L* = *xJ* for some $J\in I_{t}^{*}(H).$ Note that ${\left(J^{k}\right)}_{t}$ is principal, I⊆J and ${\left(L^{k}\right)}_{t}⊊{\left(J^{k}\right)}_{t}.$ We infer that $\lambda ({\left(J^{k}\right)}_{t})<n,$ and hence *J* is principal by the induction hypothesis. This implies that *L* is principal.
2. Let {P∈X(H)|I⊆P} be finite. Let P be the set of all finite *t*-products (which are not necessarily squarefree or nonempty) of elements of X(H). Since *H_Q_* is a DVM for each Q∈X(H) by Proposition 3.1, we infer that $\left\{C\in P|{\left(I^{k}\right)}_{t}\subseteq C\right\}$ is finite. Let $\lambda :A\to N_{0}$ be defined by λ(L)=|{C∈P|L⊆C}| for each L∈A. Now let A,B∈A be such that A⊊B. There exist x,y∈H and some nonunit z∈H such that *A* = *xH*, *B* = *yH* and *x* = *yz*. Since *H* satisfies the Principal Ideal Theorem by Proposition 3.1, there is some Q∈X(H) such that z∈Q. Moreover, there is some minimal J∈P such that yH⊆J. We have that $A=\mathrm{xH}\subseteq {\left(\mathrm{JQ}\right)}_{t}\in P$ and $B=\mathrm{yH}⊈{\left(\mathrm{JQ}\right)}_{t}⊊J$ (note that *H_Q_* is a DVM). Therefore, λ(B)<λ(A), and thus *λ* is a length function. The statement now follows by (1).
3. Let *H* be a BF-monoid, ℓ∈N and $L\in I_{t}^{*}(H)$ such that ${\left(L^{\ell }\right)}_{t}$ is principal. Set $B=\{{\left(J^{\ell }\right)}_{t}|J\in I_{t}^{*}(H),L\subseteq J,{\left(J^{\ell }\right)}_{t}$ is principal}. Since B is a subset of the set of nontrivial principal ideals of *H*, we have that B possesses a length function. Therefore, *L* is principal by (1). We infer that $C_{t}(H)$ is torsionfree. □

## 5. On the monoid of *r*-invertible *r*-ideals

In this section, we put our focus on the monoid of *r*-invertible *r*-ideals and give characterizations for this monoid to be radical factorial or to have the property that the radical of every principal ideal is principal. We also present a characterization for radical factorial monoids and discuss the connections between the monoid of *r*-invertible *r*-ideals and radical *r*-factorization of principal ideals and *r*-invertible *r*-ideals.

Lemma 5.1. *Let r be a finitary ideal system on H and* $I,J\in I_{r}^{*}(H).$

1. *I divides J in* $I_{r}^{*}(H)$ *if and only if* J⊆I.
2. *I is radical if and only if I is a radical element of* $I_{r}^{*}(H).$

Proof.

1. Let *J* be an *r*-invertible *r*-ideal of *H*. If *I* divides *J* in $I_{r}^{*}(H),$ then $J={\left(\mathrm{IA}\right)}_{r}$ for some *r*-invertible *r*-ideal *A* of *H*, and thus $J={\left(\mathrm{IA}\right)}_{r}\subseteq {\left(\mathrm{IH}\right)}_{r}=I.$ Conversely, if J⊆I, then $B={\left(JI^{-1}\right)}_{r}$ is an *r*-invertible *r*-ideal of *H* and $J={\left(\mathrm{BI}\right)}_{r},$ and hence *I* divides *J* in $I_{r}^{*}(H).$
2. First let *I* be radical, $J\in I_{r}^{*}(H)$ and k∈N such that *I* divides ${\left(J^{k}\right)}_{r}$ in $I_{r}^{*}(H).$ We have that ${\left(J^{k}\right)}_{r}\subseteq I$ by (1), and hence $J\subseteq \sqrt{J}=\sqrt{{\left(J^{k}\right)}_{r}}\subseteq I.$ Therefore, *I* divides *J* in $I_{r}^{*}(H)$ by (1).

Conversely, let *I* be a radical element of $I_{r}^{*}(H).$ Let $x\in \sqrt{I}$ be nonzero. There is some k∈N such that ${\left(\mathrm{xH}\right)}^{k}\subseteq I.$ Then *I* divides ${\left(\mathrm{xH}\right)}^{k}$ in $I_{r}^{*}(H)$ by (1), and thus *I* divides *xH* in $I_{r}^{*}(H).$ We infer that x∈xH⊆I by (1). □

Let *r* be a finitary ideal system on *H*. Next we present some (technical) characterizations of radical factorial monoids and monoids whose *r*-invertible *r*-ideals are finite *r*-products of radical *r*-ideals. Let Ω be a finite set of *r*-ideals of *H* and *I* an *r*-ideal of *H*. For each P∈X(H) let *k_P_* be the number of elements of Ω which are contained in *P*. Then Ω is called *(r, I)-meager* if for each P∈X(H) we have that $I\subseteq {\left(P^{k_{P}}\right)}_{r}.$

Proposition 5.2. *Let r be a finitary ideal system on H.*

- The following are equivalent:
  1. $I_{r}^{*}(H)$ *is radical factorial.*
  2. *Each* $I\in I_{r}^{*}(H)$ *is a finite r-product of radical r-ideals of H.*
  3. $\underset{P\in X(H)}{\cap }H_{P}=H$*, H_Q_ is a DVM for all* Q∈X(H) *and for each* $I\in I_{r}^{*}(H),\sqrt{I}=\underset{J\in \Omega }{\cap }J$ *for some (r, I)-meager set* $\Omega \subseteq I_{r}^{*}(H).$
- H is radical factorial if and only if $\underset{P\in X(H)}{\cap }H_{P}=H,$ H_Q_ is a DVM for each Q∈X(H) and for each $x\in H,\sqrt{\mathrm{xH}}=\underset{J\in \Omega }{\cap }J$ for some (t, xH)-meager set Ω of principal ideals of H.

Proof. Observe that if $\underset{P\in X(H)}{\cap }H_{P}=H,$ then g:P(H)→P(H) defined by $X_{g}=\underset{P\in X(H)}{\cap }{\left(X_{s}\right)}_{P}$ for each X⊆H is an ideal system on *H*. In particular, if $\underset{P\in X(H)}{\cap }H_{P}=H,$ then $I=\underset{P\in X(H)}{\cap }I_{P}$ for each divisorial ideal *I* of *H*.

(A) (1) ⇔ (2): Let *I* be an *r*-invertible *r*-ideal of *H*. By Lemma 5.1(1) we have that *I* is a finite *r*-product of radical *r*-ideals of *H* if and only if *I* is a finite *r*-product of *r*-invertible radical *r*-ideals of *H* if and only if *I* is a finite product of radical elements of $I_{r}^{*}(H).$ Now the statement follows easily.

(2) ⇒ (3): We infer by Proposition 3.1 that $\underset{P\in X(H)}{\cap }H_{P}=H$ and *H_Q_* is a DVM for all Q∈X(H). Let *I* be an *r*-invertible *r*-ideal of *H*. Then $I={\left(\prod_{i=1}^{n}I_{i}\right)}_{r}$ for some n∈N and finitely many radical *r*-ideals *I_i_* of *H*. Set $\Omega =\{I_{i}|i\in [1,n]\}.$ Clearly, Ω is a finite set of *r*-invertible *r*-ideals of *H* and $\sqrt{I}=\cap_{i=1}^{n}I_{i}=\underset{J\in \Omega }{\cap }J.$ Let P∈X(H) and set k=|{J∈Ω|J⊆P}|. Then $I\subseteq {\left(\prod_{J\in \Omega }J\right)}_{r}\subseteq {\left(P^{k}\right)}_{r},$ and hence Ω is an (*r*, *I*)-meager set.

(3) ⇒ (2): Claim. For each *r*-invertible *r*-ideal *I* of *H* there is some $m\in N_{0}$ such that $I⊈{\left(P^{m}\right)}_{r}$ for all P∈X(H).

Let *I* be an *r*-invertible *r*-ideal of *H*. Then there is some (*r*, *I*)-meager set Ω of *r*-invertible *r*-ideals of *H* such that $\sqrt{I}=\underset{J\in \Omega }{\cap }J.$ First we show that each element of Ω is a radical *r*-ideal of *H*. Let J∈Ω and P∈X(H).

Case 1: $\sqrt{J}⊈P.$ We have that J⊈P, and hence ${\left(\sqrt{J}\right)}_{P}=J_{P}.$

Case 2: $\sqrt{J}\subseteq P.$ Then $\sqrt{I}\subseteq P,$ and thus $P_{P}={\left(\sqrt{I}\right)}_{P}\subseteq J_{P}\subseteq {\left(\sqrt{J}\right)}_{P}\subseteq P_{P}.$ Therefore, ${\left(\sqrt{J}\right)}_{P}=J_{P}.$

Since *J* is *r*-invertible, *J* is divisorial, and since $\sqrt{J}$ is an intersection of *r*-invertible *r*-ideals of *H*, $\sqrt{J}$ is divisorial. Consequently, $\sqrt{J}=\underset{Q\in X(H)}{\cap }{\left(\sqrt{J}\right)}_{Q}=\underset{Q\in X(H)}{\cap }J_{Q}=J.$ Note that $\sqrt{I}=\sqrt{\underset{J\in \Omega }{\cap }J}=\sqrt{{\left(\prod_{J\in \Omega }J\right)}_{r}},$ and since ${\left(\prod_{J\in \Omega }J\right)}_{r}$ is *r*-finitely generated, there is some k∈N such that ${\left(\prod_{J\in \Omega }J^{k}\right)}_{r}\subseteq I.$ Set ℓ=|{J∈Ω|J⊆P}| and m=1+k|Ω|. Assume that $I\subseteq {\left(P^{m}\right)}_{r}.$ Then ${\left(\prod_{J\in \Omega }J^{k}\right)}_{r}\subseteq {\left(P^{m}\right)}_{r},$ and hence $P_{P}^{k\ell }={\left({\left(\prod_{J\in \Omega }J^{k}\right)}_{r}\right)}_{P}\subseteq {\left({\left(P^{m}\right)}_{r}\right)}_{P}=P_{P}^{m}.$ Since kℓ<m this contradicts the fact that *P_P_* is a nontrivial proper principal ideal of *H_P_*. □(Claim)

For $A\in I_{r}^{*}(H),$ we set $m_{A}=\max\{k\in N_{0}|A\subseteq {\left(P^{k}\right)}_{r}$ for some P∈X(H)} (which exists by the claim). It is sufficient to show by induction that for all $m\in N_{0}$ and $I\in I_{r}^{*}(H)$ with *m_I_* = *m*, that *I* is a finite *r*-product of radical *r*-ideals of *H*.

Let $m\in N_{0}$ and $I\in I_{r}^{*}(H)$ be such that *m_I_* = *m*. If *m* = 0, then since *I* is divisorial, $I=\underset{P\in X(H)}{\cap }I_{P}=\underset{P\in X(H)}{\cap }H_{P}=H$ and we are done. Now let *m* > 0. There is some (*r*, *I*)-meager set $\Omega \subseteq I_{r}^{*}(H)$ such that $\sqrt{I}=\underset{J\in \Omega }{\cap }J.$ As in the proof of the claim, it follows that each element of Ω is a radical *r*-ideal of *H*.

Let P∈X(H) and set ℓ=|{J∈Ω|J⊆P}|. Then ${\left({\left(\prod_{J\in \Omega }J\right)}_{r}\right)}_{P}=\prod_{J\in \Omega }J_{P}=P_{P}^{\ell }={\left({\left(P^{\ell }\right)}_{r}\right)}_{P}⊇I_{P}.$ Since *I* and ${\left(\prod_{J\in \Omega }J\right)}_{r}$ are divisorial, we have that $I\subseteq {\left(\prod_{J\in \Omega }J\right)}_{r}.$ We infer that $I={\left(L\prod_{J\in \Omega }J\right)}_{r}$ for some *r*-invertible *r*-ideal *L* of *H*. It is sufficient to show that $m_{L}<m.$ Without restriction let $m_{L}>0.$ There is some Q∈X(H) such that $m_{L}=\max\{k\in N_{0}|L\subseteq {\left(Q^{k}\right)}_{r}\}.$ Since $m_{L}>0,$ we have that I⊆L⊆Q, and thus J⊆Q for some J∈Ω. Since $I\subseteq {\left(\mathrm{JL}\right)}_{r}\subseteq {\left(Q^{m_{L}+1}\right)}_{r},$ we infer that $m_{L}<m_{L}+1\leq m.$

(B) This can be shown along the same lines as “(A) (2) ⇔ (A) (3)”, by replacing *r* with *t* and by replacing *r*-invertible *r*-ideals with nontrivial principal ideals. □

Corollary 5.3. *Let r be a finitary ideal system on H. Then H is an r-almost Dedekind r-SP-monoid if and only if H is an r-Prüfer monoid and* $I_{r}^{*}(H)$ *is radical factorial*.

Proof. Note that every *r*-almost Dedekind monoid is an *r*-Prüfer monoid and every *r*-Prüfer monoid is *r*-treed. Moreover, if every *r*-invertible *r*-ideal of *H* is a finite *r*-product of radical *r*-ideals of *H*, then clearly every nontrivial prime *r*-ideal of *H* contains an *r*-invertible radical *r*-ideal of *H*. Therefore, the equivalence is an immediate consequence of Theorem 3.9 and Proposition 5.2(A). □

Proposition 5.4. *Let r be a finitary ideal system on H. The following are equivalent:*

1. Every principal ideal of H is an r-product of finitely many pairwise comparable radical r-ideals of H.
2. The radical of every nontrivial principal ideal of H is r-invertible.
3. The radical of every r-invertible r-ideal of H is r-invertible.
4. *The radical of every principal ideal of* $I_{r}^{*}(H)$ *is principal.*
5. Every r-invertible r-ideal of H is an r-product of finitely many pairwise comparable radical r-ideals of H.

Proof. (1) ⇒ (2): This is straightforward to prove.

(2) ⇒ (3): Recall that a nontrivial *r*-ideal *J* of *H* is *r*-invertible if and only if *J_t_* is *t*-finitely generated and *J_M_* is principal for each M∈r-max(H). Let *I* be an *r*-invertible *r*-ideal of *H*. We have to show that $\sqrt{I}$ is *t*-finitely generated and ${\left(\sqrt{I}\right)}_{M}$ is principal for each M∈r-max(H). Clearly, the radical of every nontrivial principal ideal of *H* is *t*-invertible, and hence $\sqrt{I}$ is *t*-invertible by Theorem 4.2. Therefore, $\sqrt{I}$ is *t*-finitely generated. Let M∈r-max(H). Observe that the radical of every principal ideal of *H_M_* is principal, and thus ${\left(\sqrt{I}\right)}_{M}=\sqrt{I_{M}}$ is principal.

(3) ⇒ (4): Let *I* be an *r*-invertible *r*-ideal of *H*. Set $J=\sqrt{I}$ and $I=I_{r}^{*}(H).$ It is sufficient to show that $\sqrt{II}=JI$. Since I⊆J, we infer by Lemma 5.1(1) that *J* divides *I* in I, and hence II⊆JI. Since *J* is a radical element of I by Lemma 5.1(2), we have that $\sqrt{II}\subseteq JI$ Since *J* is *r*-finitely generated, there is some n∈N such that ${\left(J^{n}\right)}_{r}\subseteq I.$ Therefore, *I* divides ${\left(J^{n}\right)}_{r}$ in I by Lemma 5.1(1), and hence ${\left(JI\right)}^{n}={\left(J^{n}\right)}_{r}I\subseteq II.$ This implies that $JI\subseteq \sqrt{II}.$

(4) ⇒ (5): Let *I* be an *r*-invertible *r*-ideal of *H*. Set $I=I_{r}^{*}(H).$ By Corollary 4.5, there exist n∈N and finitely many radical elements *I_i_* of I such that $II=\prod_{i=1}^{n}I_{i}I={\left(\prod_{i=1}^{n}I_{i}\right)}_{r}I$ and $I_{i}I\subseteq I_{i+1}I$ for all i∈[1,n−1]. This implies that $I={\left(\prod_{i=1}^{n}I_{i}\right)}_{r}.$ Let i∈[1,n]. It follows by Lemma 5.1(2) that *I_i_* is a radical *r*-ideal of *H*. Furthermore, if i∈[1,n−1], then $I_{i}\subseteq I_{i+1}$ by Lemma 5.1(1).

(5) ⇒ (1): This is obvious. □

## Monoid rings and *-Nagata rings

6.

*In this section, let H always be a monoid with* z(H)=∅.

As an application, we study several ring-theoretical constructions in this section. Recall that the monoid *H* is *completely integrally closed* if for all x∈H and y∈G with $xy^{n}\in H$ for all n∈N, it follows that y∈H. Moreover, *H* is called *root-closed* if for all x∈G and n∈N with $x^{n}\in H,$ we have that x∈H. We say that *H* is a *grading monoid* if *H* is torsionless (i.e., for all x,y∈H and n∈N such that $x^{n}=y^{n}$ it follows that *x* =* y*). If not stated otherwise, we will write a grading monoid additively (from now on). Note that *H* is a grading monoid if and only if we can define a total order on it which is compatible to the monoid operation ([16, page 123]). Moreover, a nontrivial Abelian group is a grading monoid if and only if it is torsionfree. Let *R* be an integral domain, *H* a grading monoid, *K* be a field of quotients of *R* and *G* a quotient group of *H*. A sequence ${\left(x_{g}\right)}_{g\in I}$ of elements of *K* is called *formally infinite* if all but finitely many elements of that sequence are zero.

By $R[H]=R[X;H]=\{\sum_{g\in H}x_{g}X^{g}|{\left(x_{g}\right)}_{g\in H}\in R^{H}$ is formally infinite} we denote the monoid ring over *R* and *H*. It is well-known that R[H] is an integral domain. Note that R[H] is integrally closed if and only if *R* is integrally closed and *H* is root-closed [2, Theorem 3.7(d)]. Furthermore, R[H] is completely integrally closed if and only if *R* and *H* are completely integrally closed [2, Theorem 3.7(e)]. If B⊆K and Y⊆G, then set $B[Y]=\{\sum_{g\in Y}x_{g}X^{g}|{\left(x_{g}\right)}_{g\in Y}\in B^{Y}$ is formally infinite}. Let $S=\{yX^{g}|y\in R\setminus \{0\},g\in H\}$ denote the set of nonzero homogeneous elements of R[H]. Then $S^{-1}(R[H])=K[G]$ is called the homogeneous field of quotients of R[H]. It is well-known that K[G] is a completely integrally closed *t*-Bézout domain [2, Theorem 2.2]. An ideal *A* of R[H] is called homogeneous if for all formally infinite ${\left(x_{g}\right)}_{g\in H}\in R^{H}$ such that $\sum_{g\in H}x_{g}X^{g}\in A$ we have that $x_{g}X^{g}\in A$ for all g∈H (equivalently, *A* is generated by homogeneous elements of R[H]). Let *I* be an ideal of *R* and let *Y* be an *s*-ideal of *H*. Then I[Y] is a homogeneous ideal of R[H]. Also note that if *J* is an ideal of *R* and *Z* is an *s*-ideal of *H*, then I[Y]J[Z]=(IJ)[Y+Z].

Finally, note that if *R* is an integral domain, then the *t*-system on *R* and the “classical” *t*-operation on *R* coincide for nonzero ideals of *R*. More precisely, the *t*-system on *R* extends the *t*-operation on *R* to arbitrary subsets of *R*. For this reason, we do not have to distinguish between the ring theoretical and the monoid theoretical definition of “*t*” on integral domains. Since the monoid ring R[H] is an integral domain (if *H* is a (torsionless) grading monoid), these considerations also apply to R[H].

Lemma 6.1. *Let R be an integral domain, H a grading monoid, I an ideal of R, Y an s-ideal of H and A a nonzero ideal of* R[H].

1. $\sqrt[{R[H]}]{I[Y]}=\sqrt[{R}]{I}[\sqrt[{H}]{Y}].$
2. ${\left(I[Y]\right)}_{t_{R[H]}}=I_{t_{R}}[Y_{t_{H}}].$
3. *Let I be a t-ideal of R and Y a t-ideal of H. Then* I[Y] *is t-invertible if and only if I and Y are t-invertible.*
4. A=J[Z] *for some t-ideal J of R and some t-ideal Z of H if and only if A is a homogeneous t-ideal of* R[H] *if and only if A = F_t_ for some nonempty set F of nonzero homogeneous elements of* R[H].
5. *If* R[H] *is integrally closed and A is a t-ideal of* R[H] *that contains a nonzero homogeneous element of* R[H]*, then A is homogeneous.*

Proof. (1) Recall that there is some total order ≤ on *H* that is compatible with the monoid operation on *H*.

First let $f\in \sqrt{I[Y]}$ be nonzero. Then $f^{k}\in I[Y]\subseteq \sqrt{I}[\sqrt{Y}]$ for some k∈N. We have that $f=\sum_{i=1}^{n}f_{i}X^{a_{i}}$ for some $n\in N,{\left(f_{i}\right)}_{i=1}^{n}\in {\left(R^{•}\right)}^{n}$ and ${\left(a_{i}\right)}_{i=1}^{n}\in H^{n}$ with *a_j_* < *a_k_* for all j,k∈[1,n] with *j* <* k*. We show by induction on *m* that $\sum_{i=1}^{m}f_{i}X^{a_{i}}\in \sqrt{I}[\sqrt{Y}]$ for all m∈[1,n]. Let m∈[1,n]. Set $g=\sum_{i=1}^{m-1}f_{i}X^{a_{i}}.$ Then $g\in \sqrt{I}[\sqrt{Y}]$ by the induction hypothesis. We have that $\sum_{a\in H}h_{a}X^{a}={\left(f-g\right)}^{k}=\sum_{i=0}^{k}{\left(-1\right)}^{k-i}\left(\begin{array}{c} k\\ i \end{array}\right)f^{i}g^{k-i}\in \sqrt{I}[\sqrt{Y}]$ for some formally infinite ${\left(h_{a}\right)}_{a\in H}\in R^{H}.$ Note that $h_{ka_{m}}=f_{m}^{k}.$ Therefore, $f_{m}^{k}X^{ka_{m}}\in \sqrt{I}[\sqrt{Y}].$ Since fmk=0, we infer that $f_{m}^{k}\in \sqrt{I}$ and $ka_{m}\in \sqrt{Y}.$ Therefore, $f_{m}\in \sqrt{I}$ and $a_{m}\in \sqrt{Y},$ and thus $f_{m}X^{a_{m}}\in \sqrt{I}[\sqrt{Y}].$ This implies that $\sum_{i=1}^{m}f_{i}X^{a_{i}}=g+f_{m}X^{a_{m}}\in \sqrt{I}[\sqrt{Y}].$

Conversely, let $f\in \sqrt{I}[\sqrt{Y}]$ be nonzero. Then $f=\sum_{i=1}^{n}f_{i}X^{a_{i}}$ for some $n\in N,{\left(f_{i}\right)}_{i=1}^{n}\in {\left(R^{•}\right)}^{n}$ and ${\left(a_{i}\right)}_{i=1}^{n}\in H^{n}$ with *a_j_* < *a_k_* for all j,k∈[1,n] with *j* <* k*. This implies that $f_{i}\in \sqrt{I}$ and $a_{i}\in \sqrt{Y}$ for each i∈[1,n]. Consequently, there is some m∈N such that $f_{i}^{m}\in I$ and $ma_{i}\in Y$ for each i∈[1,n]. Let ${\left(m_{i}\right)}_{i=1}^{n}\in N_{0}^{n}$ be such that $\sum_{i=1}^{n}m_{i}=\mathrm{mn}.$ Clearly, there is some j∈[1,n] such that $m_{j}\geq m.$ We have that ∏i=1n(fiXai)mi=fjmjXmjaj∏i=1,i=jn(fiXai)mi∈I[Y]. Note that *f^mn^* is a sum of elements of the form $\prod_{i=1}^{n}{\left(f_{i}X^{a_{i}}\right)}^{m_{i}}$ with $m_{i}\in N_{0}$ and $\sum_{i=1}^{n}m_{i}=\mathrm{mn}.$ Therefore, $f^{\mathrm{mn}}\in I[Y],$ and hence $f\in \sqrt{I[Y]}.$

(2), (3) This follows from [7, Corollary 2.4].

(4) Let *S* denote the set of nonzero homogeneous elements of R[H]. We only need to show that if *A* = *F_t_* for some nonempty F⊆S, then A=J[Z] for some *t*-ideal *J* of *R* and some *t*-ideal *Z* of *H*. Set T={E⊆S|∅=E⊆A,|E|<∞}. Observe that $A=\underset{E\in T}{\cup }E_{v}.$ Let D∈T. By [3, Proposition 2.5] we have that *D_v_* is a homogeneous divisorial ideal of R[H]. It follows from [7, Proposition 2.5] that there exist an ideal *J_D_* of *R* and an *s*-ideal *Z_D_* of *H* such that $D_{v}=J_{D}[Z_{D}].$ Therefore, for each C∈T, there exist an ideal *J_C_* of *R* and an *s*-ideal *Z_C_* of *H* such that $C_{v}=J_{C}[Z_{C}].$ Set $J=\underset{C\in T}{\cup }J_{C}$ and $Y=\underset{C\in T}{\cup }Y_{C}.$ Note that if B,C∈T are such that B⊆C, then $J_{B}[Z_{B}]=B_{v}\subseteq C_{v}=J_{C}[Z_{C}],$ and hence $J_{B}\subseteq J_{C}$ and $Z_{B}\subseteq Z_{C}$ (since Bv={0}). Consequently, *J* is an ideal of *R* and *Y* is an *s*-ideal of *H*. Moreover, $A=\underset{E\in T}{\cup }J_{E}[Z_{E}]=J[Z].$ (Note that if x∈J[Z], then *x* can be represented as a finite sum of elements of the form $x_{b}X^{b}$ with $x_{b}\in J$ and b∈Z, and hence there is some E∈T such that all homogeneous components of *x* are in $J_{E}[Z_{E}].$) We infer that $J[Z]=A=A_{t}=J_{t}[Z_{t}],$ and thus *J_t_* = *J* is a *t*-ideal of *R* and *Y_t_* = *Y* is a *t*-ideal of *H*.

(5) Let R[H] be integrally closed and *A* a *t*-ideal of R[H] that contains a nonzero homogeneous element x∈R[H]. Let f∈A. Then there is some finite E⊆A such that $\{x,f\}\subseteq E_{v}.$ It follows from [4, Theorems 3.2 and 3.7] that *E_v_* is homogeneous. Therefore, all homogeneous components of *f* are contained in $E_{v}\subseteq A.$ □

Proposition 6.2. *Let K be a field and G a nontrivial torsionfree Abelian group. The following are equivalent:*

1. The radical of every principal ideal of K[G] is principal.
2. K[G] is radical factorial.
3. K[G] is a t-SP-domain.
4. Every nonzero prime t-ideal of K[G] contains a nonzero radical principal ideal of K[G].

If G satisfies the ascending chain condition on cyclic subgroups, then these equivalent conditions are satisfied.

Proof. The equivalence is an immediate consequence of Theorem 3.10 and Corollary 4.5. Now let *G* satisfy the ascending chain condition on cyclic subgroup. It follows from [2, Theorem 2.3(a)] that K[G] is factorial, and thus K[G] is radical factorial. □

Note that the equivalent conditions in Proposition 6.2 are not always satisfied. Let *p* be a prime number, *G* a nontrivial additive torsionfree *p*-divisible Abelian group (e.g. (G,+)=(Q,+) or $\left(G,+)=(Z[\frac{1}{p}],+\right)$) and *K* a field of characteristic *p*. Then K[G] does not satisfy the equivalent conditions in Proposition 6.2. Assume to the contrary that the radical of every principal ideal of K[G] is principal. Let a∈G be nonzero. There is some f∈K[G] such that $\sqrt{\left(1+X^{a})K[G\right]}=\mathrm{fK}[G].$ Consequently, there is some m∈N such that $f^{p^{m}}\in (1+X^{a})K[G].$ There is some nonzero b∈G such that $p^{m}b=a.$ Observe that K[G] has also characteristic *p*, and hence $1+X^{a}={\left(1+X^{b}\right)}^{p^{m}}.$ Note that $\frac{f}{1+X^{b}}$ is an element of the field of quotients of K[G]. Since K[G] is completely integrally closed, and thus root-closed, we infer that $f\in (1+X^{b})K[G].$ It follows that $\sqrt{\left(1+X^{b})K[G\right]}=\sqrt{\left(1+X^{a})K[G\right]}=\mathrm{fK}[G]=(1+X^{b})K[G].$ There is some nonzero c∈G such that *pc* = *b*. Consequently, $1+X^{c}\in \sqrt{\left(1+X^{b})K[G\right]}={\left(1+X^{c}\right)}^{p}K[G],$ and thus $1+X^{c}\in K{\left[G\right]}^{\times },$ a contradiction.

Proposition 6.3. *Let R be an integral domain, H a grading monoid and S the set of nonzero homogeneous elements of* R[H]*. The following are equivalent*:

1. R[H] *is integrally closed and every t-ideal A of* R[H] *with* A∩S=∅ *is a finite t-product of radical t-ideals of* R[H].
2. R[H] *is integrally closed and every homogeneous t-ideal of* R[H] *is a finite t-product of radical t-ideals of* R[H].
3. *Every t-ideal A of* R[H] *with* A∩S=∅ *is a finite t-product of homogeneous radical t-ideals of* R[H].
4. *Every homogeneous t-ideal of* R[H] *is a finite t-product of homogeneous radical t-ideals of* R[H].
5. R is a t-SP-domain and H is a t-SP-monoid.

Proof. (1) ⇒ (2), (3) ⇒ (4): This is obviously true.

(1) ⇒ (3), (2) ⇒ (4): This is an immediate consequence of Lemma 6.1(5).

(4) ⇒ (5): Let *I* be a nonzero *t*-ideal of *R* and let *Y* be a nonempty *t*-ideal of *H*. Then I[Y] is a homogeneous *t*-ideal of R[H] by Lemma 6.1(4). Therefore, there exist n∈N and finitely many homogeneous radical *t*-ideals *A_i_* of R[H] such that $I[Y]={\left(\prod_{i=1}^{n}A_{i}\right)}_{t}.$ It follows from Lemma 6.1 that, for each j∈[1,n], there is some radical *t*-ideal *I_j_* of *R* and some radical *t*-ideal *Y_j_* of *H* such that $A_{j}=I_{j}[Y_{j}].$ We infer that $I[Y]={\left(\prod_{i=1}^{n}I_{i}[Y_{i}]\right)}_{t}={\left((\prod_{i=1}^{n}I_{i})[\sum_{i=1}^{n}Y_{i}]\right)}_{t}={\left(\prod_{i=1}^{n}I_{i}\right)}_{t}[{\left(\sum_{i=1}^{n}Y_{i}\right)}_{t}],$ and hence $I={\left(\prod_{i=1}^{n}I_{i}\right)}_{t}$ and $Y={\left(\sum_{i=1}^{n}Y_{i}\right)}_{t}.$

(5) ⇒ (1): It follows from [19, Proposition 3.10(3)] that *R* and *H* are completely integrally closed. Therefore, R[H] is completely integrally closed, and hence it is integrally closed.

Now let *A* be a nonzero *t*-ideal of R[H] such that A∩S=∅. By Lemma 6.1 there exist a *t*-ideal *I* of *R* and a *t*-ideal *Y* of *H* such that A=I[Y]. There exist n,m∈N, finitely many radical *t*-ideals *I_i_* of *R* such that $I={\left(\prod_{i=1}^{n}I_{i}\right)}_{t}$ and finitely many radical *t*-ideals *Y_j_* of *H* such that $Y={\left(\sum_{j=1}^{m}Y_{j}\right)}_{t}.$ We infer by Lemma 6.1 that $I_{i}[H]$ is a homogeneous radical *t*-ideal of R[H] for all i∈[1,n] and $R[Y_{j}]$ is a homogeneous radical *t*-ideal of R[H] for all j∈[1,m]. Finally, we have that $A={\left(I[H]R[Y]\right)}_{t}={\left({\left(\prod_{i=1}^{n}I_{i}\right)}_{t}[H]R[{\left(\sum_{j=1}^{m}Y_{j}\right)}_{t}]\right)}_{t}={\left({\left(\prod_{i=1}^{n}I_{i}[H]\right)}_{t}{\left(\prod_{j=1}^{m}R[Y_{j}]\right)}_{t}\right)}_{t}={\left(\prod_{i=1}^{n}I_{i}[H]\prod_{j=1}^{m}R[Y_{j}]\right)}_{t}.$ □

Proposition 6.4. [cf. [19, Proposition 2.17]] *Let R be an integral domain, H a grading monoid, K a field of quotients of R and G a quotient group of H.*

1. R[H] *is a w-SP-domain if and only if R is a w-SP-domain, H is a w-SP-monoid and* K[G] *is radical factorial.*
2. R[H] *is a w-Bézout w-SP-domain if and only if R is a w-Bézout w-SP-domain, H is a w-Bézout w-SP-monoid and* K[G] *is radical factorial*.

Proof. (1) Let *R* be a *w*-SP-domain, let *H* be a *w*-SP-monoid and let K[G] be radical factorial. Note that *R* is a *t*-Prüfer domain (i.e., a P*v*MD) and *H* is a *t*-Prüfer monoid by Theorem 4.2 and Corollaries 4.4 and 5.3. Therefore, R[H] is a *t*-Prüfer domain by [4, Proposition 6.5]. In particular, if *A* and *B* are *t*-invertible *t*-ideals of R[H], then ${\left(\mathrm{AB}\right)}_{t}={\left((A\cap B)(A\cup B)\right)}_{t},$ and hence A∩B is *t*-invertible. For g∈R[H] let *C*(*g*) be the ideal of R[H] generated by the homogeneous components of *g*. Since *R* and *H* are completely integrally closed, R[H] is completely integrally closed, and hence it follows by [7, Lemmas 1.5 and 1.6] that for every nonzero $g\in R[H],\mathrm{gC}{\left(g\right)}^{-1}=\mathrm{gK}[G]\cap R[H].$ In particular, if g∈R[H] is nonzero, then $\mathrm{gR}[H]=g{\left(C{\left(g\right)}_{t}C{\left(g\right)}^{-1}\right)}_{t}={\left(C{\left(g\right)}_{t}gC{\left(g\right)}^{-1}\right)}_{t}={\left(C{\left(g\right)}_{t}(\mathrm{gK}[G]\cap R[H])\right)}_{t}$ (since R[H] is a *t*-Prüfer domain) and gK[G]∩R[H] is a *t*-invertible *t*-ideal of R[H].

By Theorem 4.2 and Corollary 4.4, it is sufficient to show that the radical of every nonzero principal ideal of R[H] is *t*-invertible. Let f∈R[H] be nonzero. It follows from Proposition 6.2 that there is some g∈R[H] such that $\sqrt{\mathrm{fK}[G]\cap R[H]}=(\sqrt{\mathrm{fK}[G]})\cap R[H]=\mathrm{gK}[G]\cap R[H]$ (here we use that K[G] is a quotient overring of R[H]). This implies that $\sqrt{\mathrm{fK}[G]\cap R[H]}$ is *t*-invertible. Moreover, $C{\left(f\right)}_{t}=I[Y]$ for some *t*-invertible *t*-ideal *I* of *R* and some *t*-invertible *t*-ideal *Y* of *H* by Lemma 6.1. It follows from Theorem 4.2 and Corollary 4.4 that $\sqrt{I}$ is a *t*-invertible *t*-ideal of *R* and $\sqrt{Y}$ is a *t*-invertible *t*-ideal of *H*. Therefore, $\sqrt{C{\left(f\right)}_{t}}=\sqrt{I}[\sqrt{Y}]$ is *t*-invertible by Lemma 6.1.

We have that $\mathrm{fR}[H]={\left(C{\left(f\right)}_{t}(\mathrm{fK}[G]\cap R[H])\right)}_{t},$ and thus $\sqrt{\mathrm{fR}[H]}=\sqrt{C{\left(f\right)}_{t}}\cap \sqrt{\mathrm{fK}[G]\cap R[H]}$ is *t*-invertible.

Now let R[H] be a *w*-SP-domain. First let y∈R be nonzero. We have that $\sqrt{\mathrm{yR}}[H]=\sqrt{\left(\mathrm{yR})[H\right]}=\sqrt{\mathrm{yR}[H]}$ is *t*-invertible by Lemma 6.1(1), Theorem 4.2 and Corollary 4.4. Consequently, $\sqrt{\mathrm{yR}}$ is *t*-invertible by Lemma 6.1(3). It follows by Theorem 4.2 and Corollary 4.4 that *R* is a *w*-SP-domain. Now let z∈H. It follows that $R[\sqrt{z+H}]=\sqrt{R[z+H]}=\sqrt{X^{z}R[H]}$ is *t*-invertible by Lemma 6.1(1), Theorem 4.2 and Corollary 4.4. Therefore, $\sqrt{z+H}$ is *t*-invertible by Lemma 6.1(3). We infer again by Theorem 4.2 and Corollary 4.4 that *H* is a *w*-SP-monoid. Finally, let f∈K[G] be nonzero. Let *S* be the set of nonzero homogeneous elements of R[H]. There is some nonzero g∈R[H] such that fK[G]=gK[G]. By Theorem 4.2 and Corollary 4.4 we have that $\sqrt{\mathrm{gR}[H]}$ is *t*-invertible, and hence $\sqrt{\mathrm{fK}[G]}=S^{-1}\sqrt{\mathrm{gR}[H]}$ is an $S^{-1}t$-invertible $S^{-1}t$-ideal of $K[G]=S^{-1}(R[H]).$ Since $S^{-1}t\leq t_{K[G]},$ this implies that $\sqrt{\mathrm{fK}[G]}$ is a *t*-invertible *t*-ideal of K[G]. Consequently, $\sqrt{\mathrm{fK}[G]}$ is a principal ideal of K[G], since K[G] is a *t*-Bézout domain.

(2) In any case, R[H] is completely integrally closed by [19, Proposition 3.10], and hence $C_{t}(R[H])≅C_{t}(R)⊕C_{t}(H)$ by [7, Corollary 2.11]. In particular, $C_{t}(R[H])$ is trivial if and only if $C_{t}(R)$ and $C_{t}(H)$ are both trivial. Therefore, the statement follows by (1) and Theorems 3.9 and 3.10. □

Let *R* be an integral domain with quotient field *K* and *H* a grading monoid with quotient group *G*. Note that if *R* is a *t*-SP-domain, *H* is a *t*-SP-monoid and K[G] is factorial, then R[H] is in general not a *t*-SP-domain.

Let *K* be a field, $G=Z^{\left(N_{0}\right)}$ (i.e., *G* is isomorphic to the free Abelian group with basis $N_{0}$) and $H=\{{\left(x_{j}\right)}_{j\in N_{0}}\in G|x_{0}\geq x_{i}\geq 0$ for all $i\in N_{0}\}.$ Note that *G* is isomorphic to the direct sum of countably many copies of Z. Clearly, *H* is a grading monoid. It follows from [20, Example 4.2] that *H* is a *t*-SP-monoid and $C_{t}(H)$ is trivial (since *H* is *t*-local). Let ${\left(X_{i}\right)}_{i\in N_{0}}$ be a sequence of independent indeterminates over *K*. Set $T=K[\{\prod_{i=0}^{\infty }X_{i}^{\alpha_{i}}|{\left(\alpha_{i}\right)}_{i\in N_{0}}\in H\}]$ and $S=K[\{X_{i},X_{i}^{-1}|i\in N_{0}\}].$ It is clear that K[H]≅T, *T* is a subring of $K[\{X_{i}|i\in N_{0}\}]$ and K[G]≅S is factorial. First we show that *T* is not radical factorial. Let $f=X_{0}^{3}{\left(X_{1}+1\right)}^{2}(X_{2}^{3}+X_{1}).$ Then $f\in T^{•}\setminus T^{\times }.$ It is sufficient to show that *f* is an atom of *T* that is not radical. Since $K[X_{1}]$ is factorial, it follows by Eisenstein’s criterion that $X_{2}^{3}+X_{1}$ is a prime element of $K[X_{1},X_{2}].$ Therefore, $X_{2}^{3}+X_{1}$ is a prime element of $K[\{X_{i}|i\in N_{0}\}].$ It is clear that *X*_0_ and $X_{1}+1$ are prime elements of $K[\{X_{i}|i\in N_{0}\}].$ Let g,h∈T be such that *f* = *gh*. Since $K[\{X_{i}|i\in N_{0}\}]$ is factorial, there are $\eta \in K^{\times },a\in \{0,1,2,3\},b\in \{0,1,2\}$ and c∈{0,1} such that $g=\eta X_{0}^{a}{\left(X_{1}+1\right)}^{b}{\left(X_{2}^{3}+X_{1}\right)}^{c}$ and $h=\eta^{-1}X_{0}^{3-a}{\left(X_{1}+1\right)}^{2-b}{\left(X_{2}^{3}+X_{1}\right)}^{1-c}.$ Without restriction let *c* = 1. Since g∈T, we infer that *a* = 3, and thus *b* = 2 (since h∈T). This implies that $h=\eta^{-1}\in K^{\times }=T^{\times },$ and hence *f* is an atom of *T*. Note that $X_{1}+1$ and $X_{2}^{3}+X_{1}$ are prime elements of *S*. Since *S* is factorial and *f* is not a square-free product of prime elements of *S*, we have that *f* is not a radical element of *S*. Since *S* is a quotient overring of *T*, we infer that *f* is not a radical element of *T*. Consequently, *T* is not radical factorial. Since *H* is completely integrally closed, it follows by [7, Lemma 2.1 and Corollary 2.10] that $C_{t}(T)≅C_{t}(H),$ and thus $C_{t}(T)$ is trivial. Therefore, if *T* is a *t*-SP-domain, then *T* is radical factorial, a contradiction.

Next we provide a simple way to construct nontrivial examples of *w*-SP-monoids (or *t*-SP-monoids) that are grading monoids (if nontrivial examples of *w*-SP-domains or *t*-SP-domains are already given). Note that if *H* is root-closed, then *H* is a grading monoid if and only if $H^{\times }$ is torsionfree. (If *H* is a grading monoid, then *H* is torsionless, and thus $H^{\times }$ is torsionfree. Now let *H* be root-closed and let $H^{\times }$ be torsionfree. Let n∈N and x,y∈H be such that *nx* = *ny*. Then n(x−y)=0∈H, and thus x−y∈H, since *H* is root-closed. We infer that $x-y\in H^{\times }.$ Since $H^{\times }$ is torsionfree and n(x−y)=0, we have that *x* =* y*. Therefore, *H* is a grading monoid.)

Remark 6.5. Let R be an integral domain, H a monoid and U a subgroup of $H^{\times }$ with U=H. Set H/U={xU|x∈H} and let V be a subgroup of $R^{\times }$ such that $R^{\times }/V$ is torsionfree (e.g. $V=R^{\times }$ or $V=\{x\in R|x^{n}=1$ for some n∈N}) and V=R•.

1. R is a w-SP-domain (resp. a t-SP-domain) if and only if $R^{•}$ is a w-SP-monoid (resp. a t-SP-monoid).
2. H is a w-SP-monoid (resp. a t-SP-monoid) if and only if H/U is a w-SP-monoid (resp. a t-SP-monoid).
3. R is a w-SP-domain (resp. a t-SP-domain) if and only if $R^{•}/V$ is a w-SP-monoid (resp. a t-SP-monoid). If these equivalent conditions are satisfied, then $R^{•}/V$ is a grading monoid.

Proof.

1. Note that $f:I_{t}(R)\to I_{t}(R^{•})$ defined by f(I)=I∖{0} for each $I\in I_{t}(R)$ is a semigroup isomorphism. Moreover, if $I\in I_{t}(R),$ then *I* is radical if and only if *f*(*I*) is radical. Therefore, the statement is an immediate consequence of Theorem 4.2 and Corollary 4.4.
2. Observe that $f:I_{t}(H)\to I_{t}(H/U)$ defined by f(I)={xU|x∈I} for each $I\in I_{t}(H)$ is a semigroup isomorphism. Furthermore, if $I\in I_{t}(H),$ then *I* is radical if and only if *f*(*I*) is radical. Again, the statement is a consequence of Theorem 4.2 and Corollary 4.4.
3. The first statement follows from (1) and (2). Set $A=R^{•}/V$ and suppose *A* is a *t*-SP-monoid. Clearly, *A* is a root-closed monoid whose elements are cancellative. Observe that $A^{\times }=R^{\times }/V$ is torsionfree. Therefore, *A* is a grading monoid. □

Let *R* be an integral domain and *X* an indeterminate over *R*. We say that * is a star operation on *R* if * is an ideal system on *R* such that d≤∗. Moreover, we say that * is a star operation of finite type if * is a finitary ideal system on *R*. Let * be a star operation of finite type on *R*. We say that *R* is a P*MD if *R* is a *-Prüfer domain. For f∈R[X] let *c*(*f*) be the content of *f*. Set $N_{*}=\{g\in R[X]|c{\left(g\right)}_{*}=R\}.$ By $\text{Na}(R,*)=\{\frac{f}{g}|f\in R[X],g\in N_{*}\}$ we denote the *-Nagata ring of *R*.

Proposition 6.6. Let R be an integral domain, * a star operation of finite type on R and X an indeterminate over R. Then R is a *-almost Dedekind *-SP-domain if and only if Na(R,∗) *is an SP-domain*.

Proof. Set S=Na(R,∗). Let I denote the monoid of *-invertible *-ideals of *R* and let J denote the monoid of invertible ideals of *S*. It follows from Corollary 5.3 that *R* is a *-almost Dedekind *-SP-domain if and only if *R* is a P*MD and I is radical factorial. Since every SP-domain is an almost Dedekind domain, it follows by analogy that *S* is an SP-domain if and only if *S* is a Prüfer domain and J is radical factorial. We infer by [9, Theorem 3.1] that *R* is a P*MD if and only if *S* is a Prüfer domain. Therefore, it is sufficient to show that if *R* is a P*MD, then the map φ:I→J defined by φ(I)=IS for all I∈I is a monoid isomorphism. Let *R* be a P*MD. It follows by [9, Lemma 2.4] that φ is a well-defined map. We continue by showing the following claim.

Claim. If *I* is a nonzero finitely generated ideal of *R*, then $I_{*}S=\mathrm{IS}.$

Let *I* be a nonzero finitely generated ideal of *R*. We have to show that $I_{*}\subseteq \mathrm{IS}.$ Let $x\in I_{*}.$ Note that $\mathrm{IS}=\{\frac{f}{g}|f\in I[X],g\in N_{*}\}.$ Since $I_{*}$ is *-invertible, we have that ${\left(II^{-1}\right)}_{*}=R,$ and hence there is some finite $E\subseteq II^{-1}\subseteq R$ such that $E_{*}=R.$ Clearly, there is some g∈R[X] such that $c(g){=}_{R}(E).$ Observe that $c{\left(g\right)}_{*}=E_{*}=R,$ and thus $g\in N_{*}.$ Moreover, $\mathrm{Ex}\subseteq II^{-1}I_{*}=I{\left(I_{*}\right)}^{-1}I_{*}\subseteq I,$ and hence Ex⊆I. Consequently, gx∈I[X]. It follows that x∈IS. □(Claim)

Now let *A* and *B* be *-invertible *-ideals of *R*. There exist nonzero finitely generated ideals *I* and *J* of *R* such that $A=I_{*}$ and $B=J_{*}.$ We infer by the claim that $\varphi ({\left(\mathrm{AB}\right)}_{*})={\left(\mathrm{AB}\right)}_{*}S={\left(\mathrm{IJ}\right)}_{*}S=\mathrm{IJS}=\mathrm{ISJS}=I_{*}SJ_{*}S=\mathrm{ASBS}=\varphi (A)\varphi (B).$ Since φ(R)=RS=S, it follows that φ is a monoid homomorphism.

To show that φ is injective, it is sufficient to show that AS∩R=A for all *-invertible *-ideals *A* of *R*. Let *A* be a *-invertible *-ideal of *R* and x∈AS∩R. There is some $g\in N_{*}$ such that gx∈A[X], and thus c(g)x⊆A. This implies that $x\in \mathrm{xR}=\mathrm{xc}{\left(g\right)}_{*}={\left(\mathrm{xc}(g)\right)}_{*}\subseteq A_{*}=A.$

Finally, we show that φ is surjective. By [9, Lemma 2.5 and Remark 3.1] we have that *S* is a Bézout domain. Therefore, we need to show that for each nonzero f∈R[X], there is some *-invertible *-ideal *A* of *R* such that φ(A)=fS. Let f∈R[X] be nonzero. Set $A=c{\left(f\right)}_{*}.$ Then *A* is a *-invertible *-ideal of *R* and it follows by [9, Lemma 2.5 and Remark 3.1] and the claim that φ(A)=AS=c(f)S=fS. □

We end this section with a remark on the power series ring.

Remark 6.7. Let R be an integral domain and X an indeterminate over R. If R⟦X⟧ is a t-Bézout t-SP-domain, then R is a t-Bézout t-SP-domain.

Proof. Let R⟦X⟧ be a *t*-Bézout *t*-SP-domain. By Corollary 4.5 we have to show that the radical of every principal ideal of *R* is principal. Let x∈R. By Corollary 4.5 there is some g∈R⟦X⟧ such that $\sqrt[{R⟦X⟧}]{\mathrm{xR}⟦X⟧}=\mathrm{gR}⟦X⟧.$ Let *g*_0_ be the constant coefficient of *R*. It is sufficient to show that $\sqrt{\mathrm{xR}}=g_{0}R.$ We have clearly that $\sqrt{\mathrm{xR}}\subseteq \sqrt[{R⟦X⟧}]{\mathrm{xR}⟦X⟧}=gR⟦X⟧.$ Consequently, if $f\in \sqrt{\mathrm{xR}},$ then *f* = *gh* for some h∈R⟦X⟧, and hence $f=g_{0}h_{0}\in g_{0}R.$ To prove the converse inclusion, observe that $g^{k}=\mathrm{xy}$ for some k∈N and y∈R⟦X⟧. Therefore, $g_{0}^{k}=xy_{0}\in \mathrm{xR},$ and thus $g_{0}\in \sqrt{\mathrm{xR}}.$ □

Note that the converse of Remark 6.7 is not true, since there is a factorial domain *S* for which S⟦X⟧ is not factorial (as shown in [21]). Clearly, *S* is a *t*-Bézout *t*-SP-domain and a Krull domain. Therefore, S⟦X⟧ is a Krull domain as well, but it fails to be a *t*-Bézout domain, since a *t*-Bézout Krull domain is obviously a factorial domain.
